# Nanoscale modifications in the early heating stages of bone are heterogeneous at the microstructural scale

**DOI:** 10.1371/journal.pone.0176179

**Published:** 2017-04-19

**Authors:** Aurélien Gourrier, Céline Chadefaux, Estelle Lemaitre, Ludovic Bellot-Gurlet, Michael Reynolds, Manfred Burghammer, Marie Plazanet, Georges Boivin, Delphine Farlay, Oliver Bunk, Ina Reiche

**Affiliations:** 1 Université Grenoble Alpes, Laboratoire Interdisciplinaire de Physique (LIPHY), Grenoble, France; 2 CNRS, LIPHY, Grenoble, France; 3 European Synchrotron Radiation Facility, Grenoble, France; 4 Sorbonne Universités, Université Paris 06, Laboratoire d’Archéologie Moléculaire et Structurale, UMR 8220 CNRS, Paris, France; 5 Sorbonne Universités, MONARIS "De la molécule aux nano-objets: réactivité, interactions et spectroscopies", UMR 8233 UPMC-CNRS, Université Pierre et Marie Curie Paris 6, Paris, France; 6 LYOS, INSERM U1033, Lyon, France; 7 Swiss Light Source, Paul Scherrer Institut, Villigen PSI, Switzerland; 8 Rathgen Forschungslabor, Staatliche Museen zu Berlin Stiftung Preußischer Kulturbesitz, Berlin, Germany; Indiana University Purdue University at Indianapolis, UNITED STATES

## Abstract

Nanoscale studies of bone provide key indicators to evidence subtle structural changes that may occur in the biomedical, forensic and archaeological contexts. One specific problem encountered in all those disciplines, for which the identification of nanostructural cues could prove useful, is to properly monitor the effect of heating on bone tissue. In particular, the mechanisms at work at the onset of heating are still relatively unclear. Using a multiscale approach combining Raman microspectroscopy, transmission electron microscopy (TEM), synchrotron quantitative scanning small-angle X-ray scattering imaging (qsSAXSI) and polarized light (PL) microscopy, we investigate the ultrastructure of cortical bovine bone heated at temperatures < 300°C, from the molecular to the macroscopic scale. We show that, despite limited changes in crystal structure, the mineral nanoparticles increase in thickness and become strongly disorganized upon heating. Furthermore, while the nanostructure in distinct anatomical quadrants appears to be statistically different, our results demonstrate this stems from the tissue histology, i.e. from the high degree of heterogeneity of the microstructure induced by the complex cellular processes involved in bone tissue formation. From this study, we conclude that the analysis of bone samples based on the structure and organization of the mineral nanocrystals requires performing measurements at the histological level, which is an advantageous feature of qsSAXSI. This is a critical aspect that extends to a much broader range of questions relating to nanoscale investigations of bone, which could also be extended to other classes of nanostructured heterogeneous materials.

## Introduction

Identifying the precise impact of heating on bone constitutes a challenge for archaeological and forensic sciences [1–3]. While traces of heating can provide valuable clues on past socio-cultural behaviors [4–6], the use of heated samples for biochemical analysis as, e.g., radiocarbon 14C dating [7] or DNA extraction [8] can also introduce an analytical bias. Similarly, in medico-legal practice, quantitative data are often lacking when a precise determination of the temperature, duration of heating or the physical nature of the heat source is required [9]. Although serving different purposes, such examples are closely related in that the detailed mechanisms of the heating processes and their induced structural and chemical modifications must be deciphered with a high degree of precision. The experience gained in those fields of research shows that: 1) this is only possible using models based on modern samples heated under well-controlled conditions and 2) that macroscopic and microscopic markers are often insufficient, hence the search for nanoscopic ones [10].

To meet this end, it is essential to consider the insight gained from structural studies in the biomedical field. The hierarchical structure of bone and its composite nature at the nanoscale have been recognized as the two main determinants of its biomechanical properties [11–14]. Thus, the structure of the hydrated collagen microfibrils of ~ 100 nm in diameter reinforced by mineral nanocrystals of ~ 5 × 50 × 100 nm^3^ and the collective microfibril organization appear as key features that could be altered by heating. Micro-thermal, differential scanning calorimetry (DSC), transmission electron microscopy (TEM) and Fourier transformed infrared (FTIR) spectroscopy investigations have, indeed, shown that the organic matrix undergoes a series of structural modifications before degradation at ~ 400°C [6,15–18]. Such changes are well correlated with macroscopic weight loss, volume shrinkage and decrease in hardness [19]. Interestingly, many studies undertaken with X-ray diffraction (XRD) conclude that the mineral nanocrystals size and structure are not modified before ~ 400–600°C, above which coalescence and recrystallization phenomena occur [19–21]. Such effects were confirmed by scanning electron microscopy (SEM) [22] and were used to estimate the heating temperature in archaeological contexts [21,23–24]. However, that relatively important structural changes may occur in the organic phase at temperatures < 400°C without affecting the mineral nanocrystalline structure and organization is conceptually difficult to reconcile with our current understanding of the intimate connection existing between the two phases at the nanoscale [25]. Most recent biomineralization studies rather highlight the importance of the organic matrix in the formation of the mineral nanocrystals [26]. Furthermore, several authors have pointed to the presence of a hydrated layer at the surface of the mineral nanocrystals [27–28] which should be strongly affected by heat.

Our work hypothesis is that the mineral nanostructure may well experience subtle changes that could only be detected using an integrative multimodal approach. Recently, an extended combination of methods including SEM, TEM and DSC provided evidence of an increase in mineral nanocrystal size and a perturbation in their organization concomitant with collagen structural modifications [10]. However, a precise quantification of those parameters was limited to very small sample regions (~ 1–10 μm^2^) typically accessible by TEM with a spatial resolution high enough to visualize the individual crystals (~ 2 nm). This is problematic due to the high degree of structural heterogeneity at the microscopic level (in the 100 μm range) which arises from the biological processes involved in bone formation and remodeling. The diversity of histological features encountered within a macroscopic bone sample, which forms the basis of histology [29], can be seen as a strongly heterogeneous microstructure in terms of material science. This constitutes an additional degree of complexity since this microstructural heterogeneity, which is of primary importance in biomedical studies, needs to be taken into account when studying the properties of bone at the nanoscale. In practice, however, the nanoscale information is, often, either obtained as an average parameter of the whole macroscopic or microscopic structure, as with XRD or FTIR, or within a very localized sample volume which is assumed to be representative of the whole tissue, as with TEM. In this paper, we revisit this issue using a multiscale approach focused on changes of the mineral nanostructure from the atomic to the macroscopic scale in a well-defined bovine cortical bone model heated under controlled laboratory conditions. Using position-resolved measurements which allow distinguishing the individual histological features, we show how the structural changes at the nanoscale can be interpreted in the light of the microstructural complexity, thus taking into account the fluctuations induced by the biological processes of bone formation and remodeling.

## Materials and methods

### Bone sections

The samples were prepared from a bovine tibia obtained from the local slaughterhouse, 38120 Fontanil-Cornillon, France. The periosteum and marrow were mechanically removed and a transverse cross-section of ~ 15 mm in thickness was sawed in the diaphysis, fixed with ethanol 70%_v_, dehydrated in a graded series of ethanol solutions of 80%_v_, 90%_v_ and 100%_v_. Cortical blocks of ~ 10 × 10 × 10 mm^3^ were selected in the anterior (A) and posterior (P) quadrants and 14 adjacent transverse sections of 200 μm in thickness were cut from each block using a high precision low-speed diamond saw (Accutome 5, Struers). 2 × 7 sections (A and P) were selected for the μRaman measurements and the remaining 2 × 7 for qsSAXSI experiments. Both subsets were reduced in thickness down to 80 ± 2 μm for the Raman samples and 60 ± 2 μm for the qsSAXSI samples, by polishing with high grade SiC paper (2400). For each series of 7 samples, 1 section was kept as a reference while the 6 remaining were heated to 100°C, 150°C, 170°C, 190°C, 210°C, 250°C in air during 1 h in a furnace. These temperatures were selected to cover the range between ambient temperature and 250°C, i.e. before the initial stages of collagen degradation beyond which SAXS analysis becomes impractical. The choice of finer steps between 170°C and 210°C were based on previous DSC, FTIR and TEM results pointing to important structural changes at the sub-microscopic level around 190–200°C [10]. The electron microscopy samples were obtained from the remaining parts of the posterior block. One sample was kept as reference while the other two were heated at 170°C, 190°C and 200°C for 1 h. The block was trimed with a glass knife in order to reduce the surface and cut without embedding by ultramicrotomy (Reichert Ultracut E) using a diamond knife (Diatom 35°). Ultrathin sections of 70 nm were deposited on 200-mesh copper grids coated with a membrane of carbon (Formwar).

### Light microscopy (LM) and image registration

The samples were imaged by direct and crossed polarized light (PL) microscopy in transmission mode (Olympus IX71) using a 10× air objective (0.30 NA) and a Canon EOS 70D camera. Mosaic LM images of the full samples and their combination with the qsSAXSI images were reconstructed using ImageJ [30].

### Micro-raman measurement and analysis

Raman measurements were performed using an inVia Raman micro-spectrometer (Renishaw) equipped with a near-infrared laser (*λ* = 785 nm). Position-resolved measurements were acquired with a focal spot of 8 μm in diameter (20× magnification with 0.4 NA) using 50% of the initial laser power of 300 mW for 30 s integration time. A 1200 lines.mm^-1^ grating was used in extended mode allowing to span a spectral range of 100–3200 cm^-1^. For each sample, 10 measurements were made within well identified regions of the microstructure with lamellar or fibrous collagen organization, based on comparison with polarized light microscopy images. The Raman spectra obtained at 250°C was decomposed using wavelet analysis (Daubechies 11 functions) using a custom script written in Python and the first 7 levels were used to subtract the background by least square fitting to all other spectra, similar to the method described by Ramos et al. [31]. The major peaks of the spectra from the organic and mineral phases were fitted using Gaussian functions within the following spectral regions: 370–650 cm^-1^, 750–1140 cm^-1^, 1140–1550 cm^-1^ and 2800–3100 cm^-1^. In addition to the analysis of peak shifts, the ratio of the integrated intensity (peak area) of the I(ν_1_PO_4_)/I(νAmideI) and the I(ν_1_PO_4_)/I(νCH) bands provide an estimation of the mineral/organic fraction, while the I(νCO_3_)/I(ν_1_PO_4_) ratio relates to the relative content of carbonate and phosphate groups in the mineral nanocrystals [32–34].

### TEM measurements and analysis

TEM observation was carried out with a Philips EM208 microscope operating at 80 kV with a maximum spatial resolution of ~ 0.1 nm at the Centre Commun de Micorscopie Electronique (CCME), Orsay, France. The particle thickness was estimated by analyzing intensity profiles measured at positions in which the mineral nanoparticles were clearly distinguishable. The derivative of the intensity profiles was calculated and fitted using gaussian functions. The full-width at half maximum (FWHM) of each gaussian was taken as the local particle thickness estimate.

### Synchrotron scanning-SAXS

The scanning-SAXS experiments were performed at the cSAXS beamline of the Swiss Light Source (Paul Scherrer Institut, Villigen, Switzerland) using a monochromatic X-ray beam of wavelength of *λ* = 0.667 Å (*E* = 18.58 keV) focused to 50(H) × 20(V) μm^2^ (FWHM) at sample position with a photon flux of 5 × 10^10^ ph.s^-1^. The samples were mounted between Kapton® foils (25 μm thick) and scanned over the full cortical thickness (~ 11.5 mm) and 1.5 mm across with a step size of 50(H) × 20(V) μm^2^. To obtain a larger view of the nanoscale changes, the reference sample in the posterior quadrant was analyzed over a larger region of 10(H) × 10(V) mm^2^ with a step size of 30(H) × 20(V) μm^2^. The parasitic scattering was reduced using a 2.1 m evacuated flight tube between the sample and the detector. SAXS patterns were collected with an exposure time of 30 ms using a Pilatus 2M detector at full resolution (1475 × 1679 pixels of 172 × 172 μm^2^). This configuration provided a measurable *q*-range of 0.01–4.1 nm^-1^ where *q* is the norm of the scattering vector *q* = 4πsin*θ*/*λ* and *θ* is the scattering angle.

The two dimensional SAXS patterns were azimuthally integrated using the FIT2D software package [35]. The one dimensional profiles were subsequently analyzed using a dedicated SAXS analysis library written in Python. Several structural parameters relating to the mineral nanoparticle size, lateral organization and orientation can thus be determined following procedures established by Fratzl *et al*. for bone studies, see, e.g. [36]. The average chord length, the so-called *T* parameter, can be considered as a standard parameter in the SAXS analysis of bone in both the medical [37–38] and the archaeological context [39–41]. It represents the ratio of the product of volume fractions of mineral *Φ* and organic (1 –*Φ*) to the total interface per unit volume of tissue *σ*:

$$T=\frac{4\emptyset (1-\emptyset )}{\sigma }$$ (1)

Under the assumption of a 50% volume fraction of mineral phase, Fratzl et al. showed that *T* gives a direct measure of the thickness for platelet-shaped nanoparticles. In the present work, *T* was calculated using a recent method based on extensive electron microscopy evidence showing that the mineral phase can be described, to a good approximation, in the form of stacks of platelet-shaped nanoparticles embedded within the collagen matrix. This method involves fitting the profile *q*^2^*I*(*q*) vs *q*, where *I*(*q*) is the azimuthally integrated scattered intensity, with an analytical expression containing three independent parameters: *T*, *α* and *β* [42–43].

$$q^{2}I(q)=PT^{2}\frac{q^{2}T^{2}+(\alpha -1)(\alpha^{2}+\beta^{2})}{q^{4}T^{4}+2(\alpha^{2}-\beta^{2})q^{2}T^{2}+{\left(\alpha^{2}+\beta^{2}\right)}^{2}}$$ (2)

The so-called Porod constant *P* is calculated independently as:

$$P=\lim_{q\to \infty }\left(q^{4}I(q)\right)$$ (3)

In this model, 2π/*α* provides an indication of the relative extent of the ordering, lower values for this quantity pointing towards a greater disorder. Similarly, *d* = *T*.2π/*β* describes the typical inter particle distance between particles. In this way quantitative maps of the structural parameters of interest as a function of scan position can be reconstructed [44–47]. This procedure is referred to as quantitative scanning-SAXS imaging (qsSAXSI) in this manuscript.

### Statistical analysis

Differences between data sets were assessed using non-parametric Mann Witney U tests using the stats module of the scipy Python package [48] and R software [49]. p < 0.05 was considered to be significant (and p < 0.01 highly significant). A 90% confidence interval for the difference between two data sets was also calculated when significant. The degree of correlation between two variables was assessed by calculating the Spearman correlation coefficient ρ.

## Results

### Temperature evolution of the mineral and organic phase at the molecular scale

**Fig 1:**
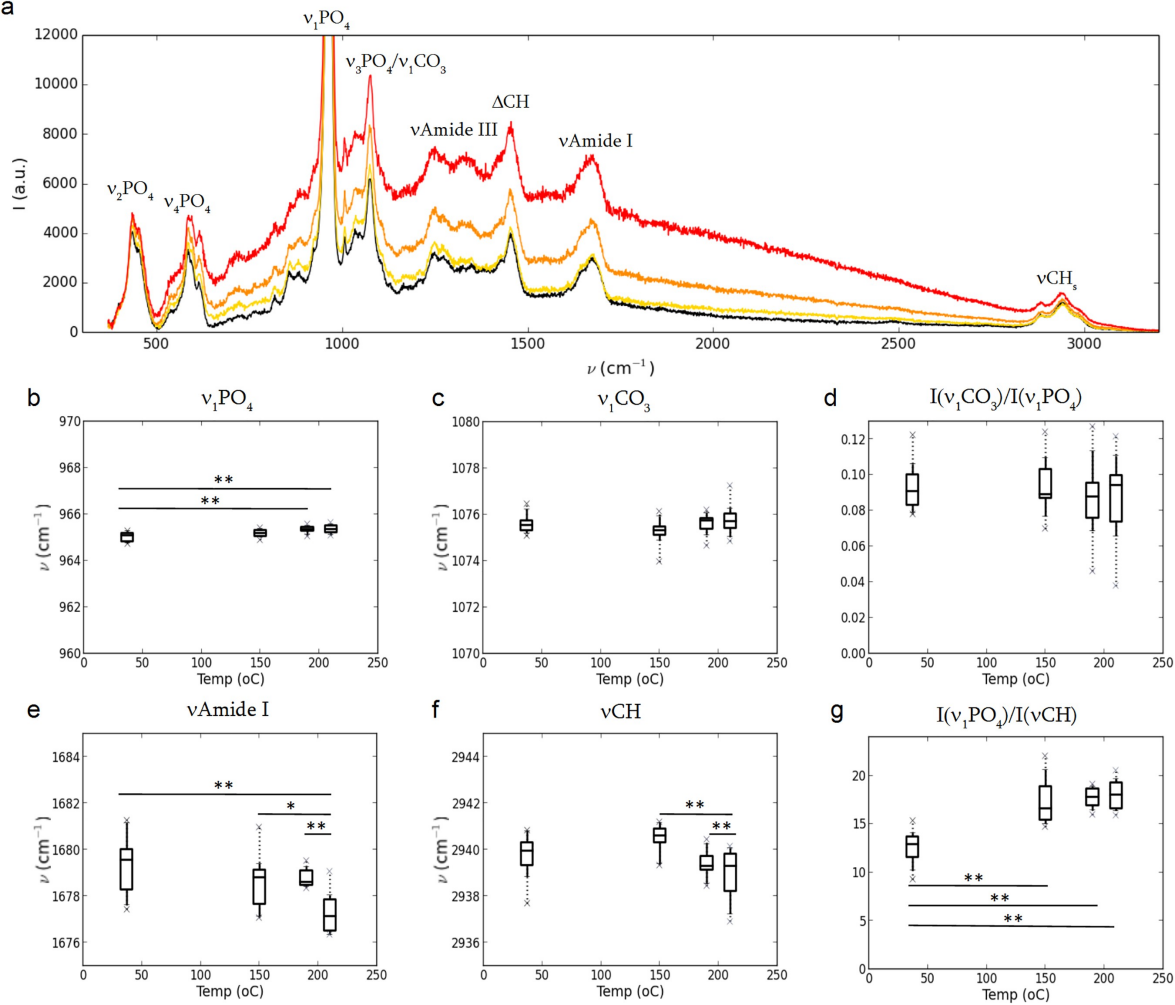
Effect of heating at the molecular scale. (a) Average Raman spectra for the posterior section for the reference sample (black), 150°C (yellow), 190°C (orange) and 210°C (red) with the main vibrational band attributions. The structural effect of heating is assessed by changes in band positions of the mineral phase: (b) phosphate ν_1_PO_4_, (c) carbonate ν _1_CO_3_ and of the organic matrix: (e) νAmideI, (f) νCH. All band positions are displayed within the same interval (Δν = 10 cm^-1^). Mineral compositional changes are evaluated by the carbonate/phosphate ratio (d) I(ν_1_CO_3_)/I(ν_1_PO_4_), while the volume ratio of the mineral/organic fraction is provided by (g) I(ν_1_PO_4_)/I(νCH).

In order to assess the extent of possible changes of the organic phase at the molecular level and of the crystal structure of the mineral nanoparticles, a series of Raman spectroscopy measurements were performed on heated samples. The vibrational bands can be observed at all temperatures up to 210°C (Fig 1A) indicating that, at the molecular scale, both the mineral and the organic structures are at least partly preserved. However, a broad background can also be observed, which is generally attributed to fluorescence from the organic matrix. This background increases very strongly between 190–210°C up to a point where it was impossible to collect a spectrum with meaningful signal/background ratio at 250°C (S1 Fig).

Two aspects were analyzed: 1) the shift in peak positions of specific bands, which indicates changes in vibration modes of specific molecular groups, and thus of the internal structure of the organic or the mineral phase, and 2) the relative changes in band intensities between organic and mineral phases which provide indications on the volume fractions ratio.First, it can be noted that the positions of the main peaks of the phosphate (ν_1_PO_4_) and the carbonate (ν_1_CO_3_) groups (Fig 1B and 1C) are relatively narrowly distributed (95% data within Δν_1_PO_4_ = 0.83 cm^-1^ at maximum (reference sample) and within Δν_1_CO_3_ = 2.61 cm^-1^ at most (at 210°C). Significant shifts in the ν_1_PO_4_ band position were only detected between ambient and 190–210°C with an estimated difference of Δν = 0.15–0.48 cm^-1^ (p < 0.01, 90% confidence interval; see S1 Table). Due to a larger spread in data, no significant changes were observed in the ν_1_CO_3_ position (S2 Table). Interestingly, the carbonate to phosphate ratio (Fig 1D), remains comparable at all temperatures, pointing to an overall conservation of mineral chemical composition. On the contrary, the vibrational bands attributed to the organic matrix are more broadly distributed and significant changes can be observed: first, the νAmide I band attributed to collagen shifts towards lower wavenumbers with an estimated maximum difference of Δν = -3.22 –-1.11 cm^-1^ between ambient and 210°C (Fig 1E; p < 0.01 between the reference, 150°C and 210°C and p < 0.05 between 150°C and 190°C; see S3 Table); secondly, the νCH stretch arising from both the collagen and non-collagenous proteins [32] is stable between ambient and 150°C but also shifts significantly between 150–210°C with an estimated maximum difference of Δν = -2.36 –-0.75 cm^-1^ (Fig 1F; p < 0.01; see S4 Table). Furthermore, the I(ν_1_PO_4_)/I(νCH) and the ν_1_PO_4_/ νAmide I (not shown) intensity ratio increase as a function of temperature but are stable above 150°C (Fig 1G; S6 Table), suggesting that the changes of the mineral to organic volume ratio mostly occur in the early stages of heating.

The mechanisms of collagen denaturation upon heating have been thoroughly investigated since the 70s concluding to a progressive protein denaturation at temperature as low as 42°C (see, e.g. [50]) which was attributed to the presence of thermally labile domains in the collagen sequence [51]. However, early studies using XRD also reported that mature collagen in fully mineralized bone does not degrade significantly up to 90–100°C (contrary to demineralized bone heated under the same conditions), thus emphasizing the protective effect of the mineral phase intricately associated to the collagenous matrix [52]. This has, since, been confirmed by DSC and electric conductivity analysis which identified two transitions on dried bovine femoral cortical bone at 122°C and within 156–182°C corresponding, respectively, to a progressive evaporation of loosely and tightly bound water and to a progressive unwinding of the collagen triple helix [53]. More recent microthermal measurements also reported such transitions at 130°C and 170–180°C [17,54].

Based on those observations, we can postulate that the significant change between ambient and 150°C observed for the I(ν_1_PO_4_)/I(νCH) ratio is related to tightly bound water, with a concomitant change in collagen structure pointed by the νAmide I shift. The absence of significant variation of the νCH band position within this temperature range suggests that this structural modifications remain limited up to 150°C. This denaturation becomes more important above 150°C as shown by the shifts of both organic bands. Nevertheless, the observation of all bands at temperatures < 210°C and the relative stability of the I(ν_1_PO_4_)/I(νCH) ratio between 150–210°C strongly suggest that the collagen is not fully denatured in this range. However, the absence of measurable Raman spectra at 250°C as a consequence of an increased background tends to indicate that the full denaturation and even degradation occurs in our samples between 210–250°C, as previously reported by thermal analysis [17] X-ray diffraction [52] and FTIR [18].

### Dimensional analysis of the effect of heating on the mineral nanoparticles

#### A localized TEM analysis

**Fig 2:**
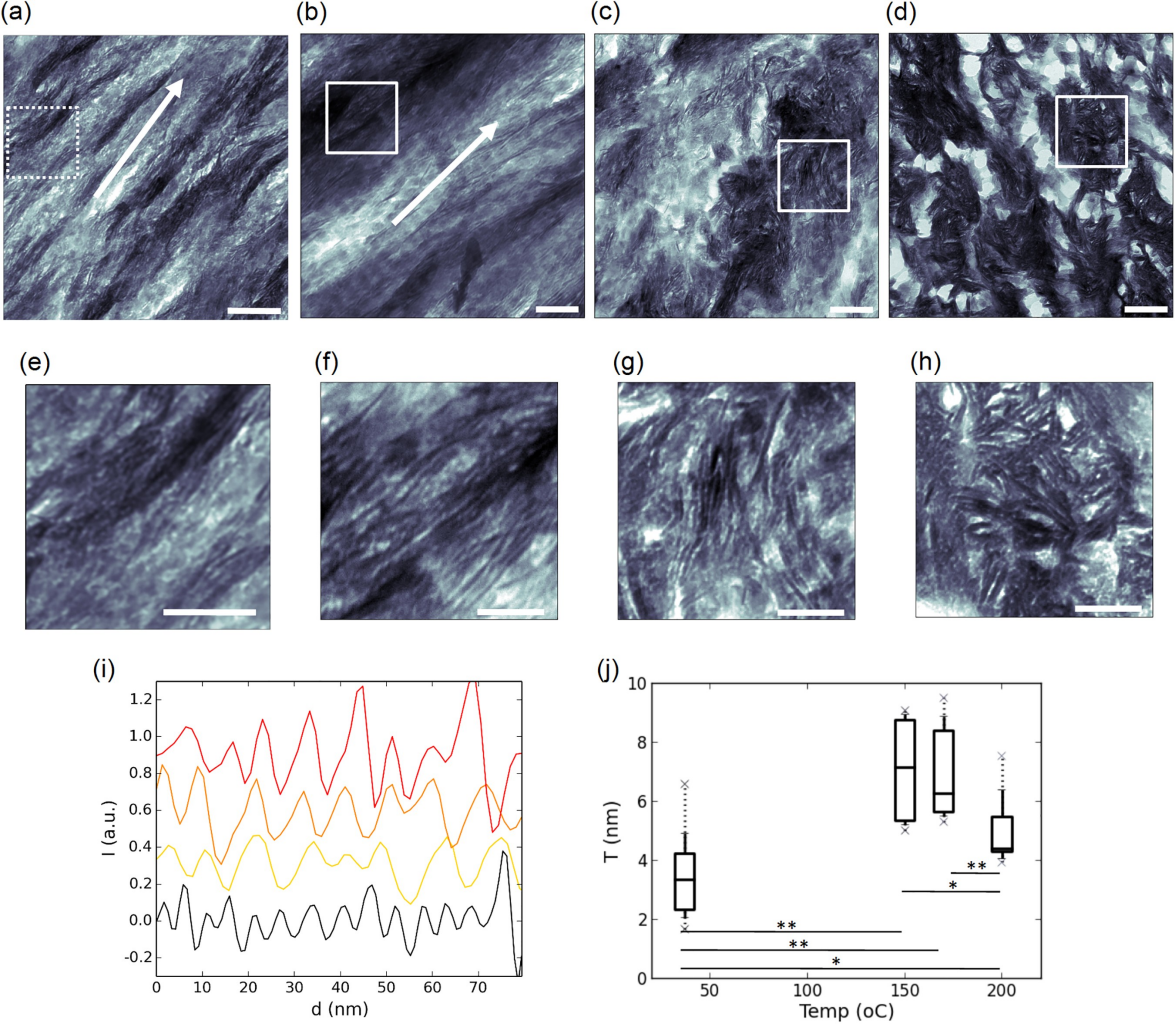
Progressive disorganization of the mineral/matrix nanoscale structure upon heating. TEM images of the reference sample (a) and samples heated at 150°C (b), 170°C (c) and 200°C (d). A higher resolution image of the region of the reference sample indicated by a dashed white rectangle is shown in (e). Enlarged views of the white rectangle regions of the heated samples are shown in (f,g,h). The derivative of the intensity profiles of the four samples are shown in (i) with the same color code as Fig 1A and the corresponding thickness distribution of the particles is shown in (j). Scale bar: 100 nm in (a-d) and 400 nm in (e-f).

Transmission electron microscopy (TEM) images were acquired on a reference sample (Fig 2A) and samples heated at 150°C (Fig 2B), 170°C (Fig 2C) and 200°C (Fig 2D), which were previously identified as key figures for low temperature modifications of bone ultrastructure [10]. A typical banding pattern of collagen is observed in the reference sample and the one heated at 150°C in the form of alternating dark and bright layers along the white arrow in Fig 2A and 2B. This pattern is known to be related to the regular packing of the collagen molecules into dense microfibrils with a shift along the collagen axis leading to the presence of gaps and, therefore, to low density regions appearing bright on the image with a period of 67 nm [55–57]. The presence of this periodic motif strongly supports the limited changes in supramolecular structure postulated in the Raman analysis. Similarly, the loss of this feature above 150°C also points to a more pronounced perturbation of the collagen microfibril structure.

Additionally, thin, elongated mineral nanoparticles, appearing dark in Fig 2E, 2F, 2G and 2H, have previously been shown to be platelets observed in cross section at different angles [58–61]. Thus, the nanoparticles oriented along the direction of observation appear well separated, as in the regions shown in Fig 2E, 2F, 2G and 2H, while those that are tilted with respect to the path of the electron beam may overlap in the projected image and are therefore more complicated to distinguish. However, the nanoparticles remain approximately with same shape, i.e. thin and elongated and the most important difference between the images is the progressive disorganization of the mineral nanoparticles which orientation ranges from highly uniform in Fig 2A and 2B to totally random in Fig 2C and 2D. Because the length and width of the mineral platelets is difficult to estimate from the TEM images due to the orientation effects, the most precise metric to characterize the mineral nanoparticles is their thickness. Furthermore, due to the inhomogeneities in gray levels between the mineral phase (darker), the organic phase (bright) and regions where there is no material (very bright) and to the limited spatial resolution of the images, a direct segmentation based on the gray levels histogram was not possible. Thus, the thickness was assessed from line profile intensities in regions of the images where the particles are best separated (Fig 2I). The distribution of thickness as a function of temperature is shown in Fig 2J. All particle sizes distribution were found to differ significantly (see S7 Table) except between 150 and 170°C. A two-fold increase is observed between the reference sample and those heated at 150 and 170°C albeit with a broader distribution in size. A significant decrease can be noted at 200°C but the estimation was more complicated due to a strong particle overlap.

#### Global SAXS analysis

**Fig 3:**
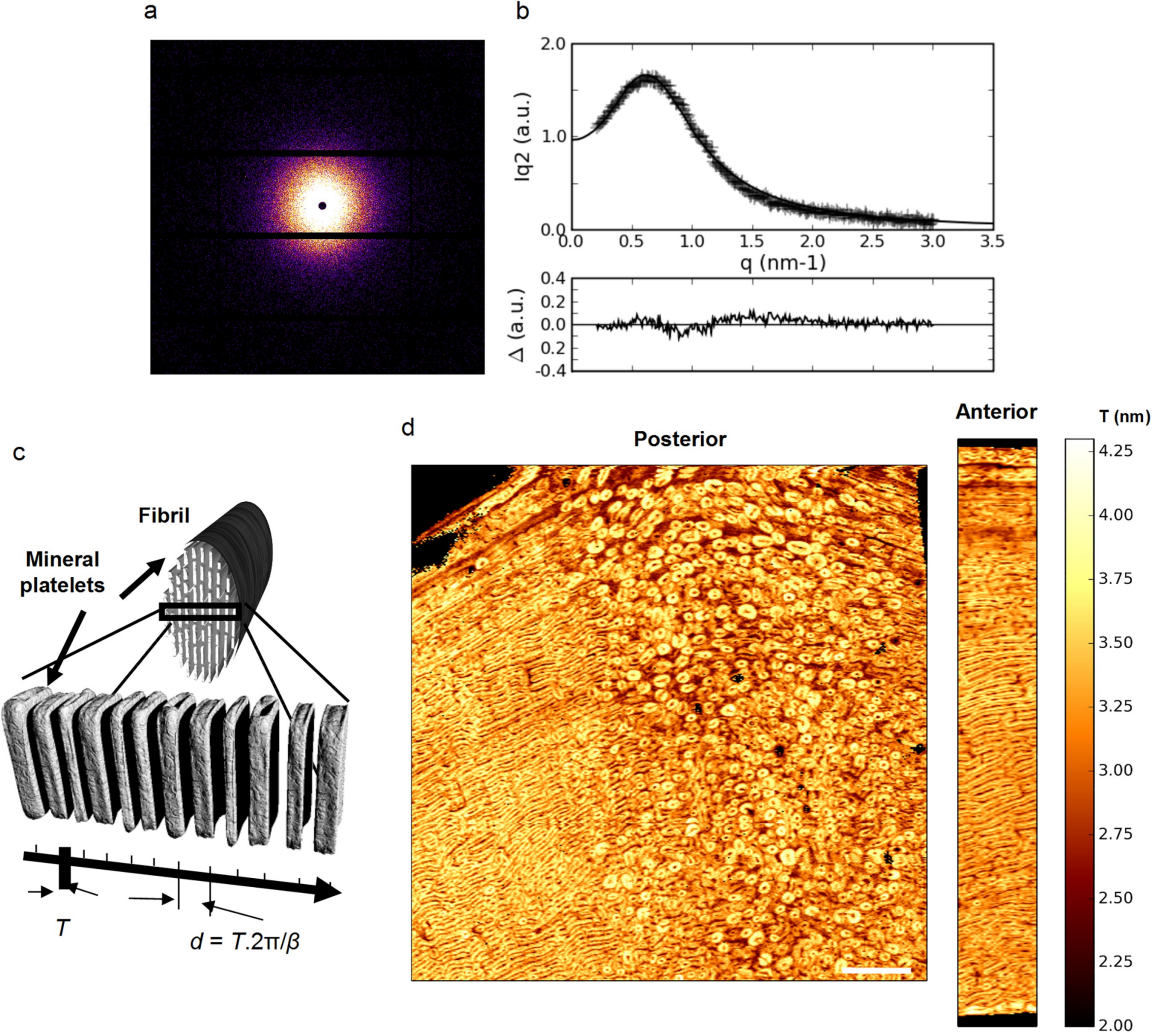
Mineral nanoscale distribution in the reference samples. (a) representative 2D SAXS image and (b) its corresponding 1D radial profile fitted using the model schematically described in (c). (d) qsSAXSI images of *T* (nm) for the posterior and anterior sections. The qsSAXSI images are displayed on the same color and size scale for comparison. Scale bar: 1,5 mm.

A typical 2D SAXS pattern and its corresponding 1D radial intensity profile are shown in Fig 3A and 3B respectively. The fitting parameters of the 1D profile provide estimates of the average SAXS correlation length (*T*), which represents the particle thickness in first approximation (Fig 3C), of the short range order (2π/*α*) and of the degree of regularity (2π/*β*) according to Eq 2 [42–43]. The images representing *T* as a function of scan position for the reference posterior and anterior sections are shown in Fig 3D.

**Fig 4:**
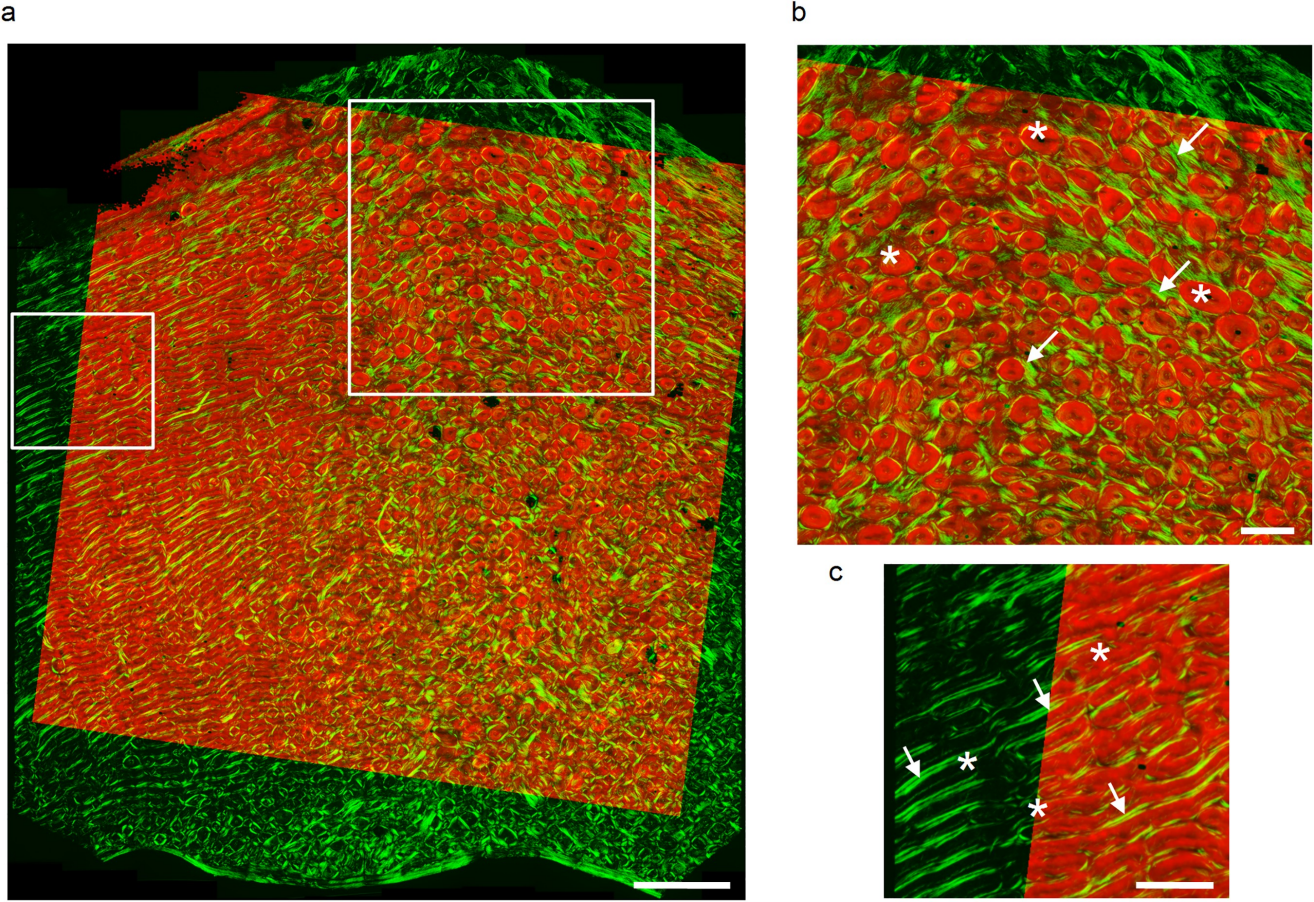
Site-matched correspondence between the tissue microstructure and the nanocrystal thickness. (a) overlay of the polarized light microscopy image (green) and the qsSAXSI image of T (red). The large box in (a) is magnified in (b) showing osteonal bone, where the osteons are indicated by stars and interstitial tissue by arrows. The smaller box in (a) is enlarged in (c) and shows fibrolamellar bone formed by an alternating sequence of lamellar (arrows) and fibrous (stars) tissue. Scale bars: 1,5 mm in (a) and 0,5 mm in (b,c).

A first striking observation, is the appearance in the qsSAXSI image of the posterior reference sample (Fig 3D) of a positive contrast revealing a high degree of structural heterogeneity closely matching the polarized light (PL) microscopy image of the sample (Fig 4A; S2 Fig). In the right part of this image, cylindrical features closely resembling osteons (~ 200 μm in diameter) can be found, surrounded by interstitial tissue. Additionally, in the left part of the reference sample, a pattern of alternating lamellar and fibrous bone packets can be observed, generally referred to as fibrolamellar bone [12] which is morphologically very similar to the microstructure of the anterior reference sample which was found to be much more homogeneous (hardly any osteons at all) under PL examination.

This strongly suggests that the nanoscale characteristics of the mineral phase in bone are correlated with the local microstructure. The positive contrast of Fig 3D indicates higher *T* values for the osteons in comparison with the interstitial tissue. The fact that the merged image in Fig 4A is dominantly red or green (and not intermediate colors) emphasizes the anti-correlation between the *T* image and the PL one, in particular in the osteonal tissue (Fig 4B). The situation is less clear in the fibrolamellar bone (Fig 4C), but the lamellar regions where the collagen appears most organized (bright green) seem to correspond to lower values of *T* and fibrous tissue (dark) to higher ones.

**Fig 5:**
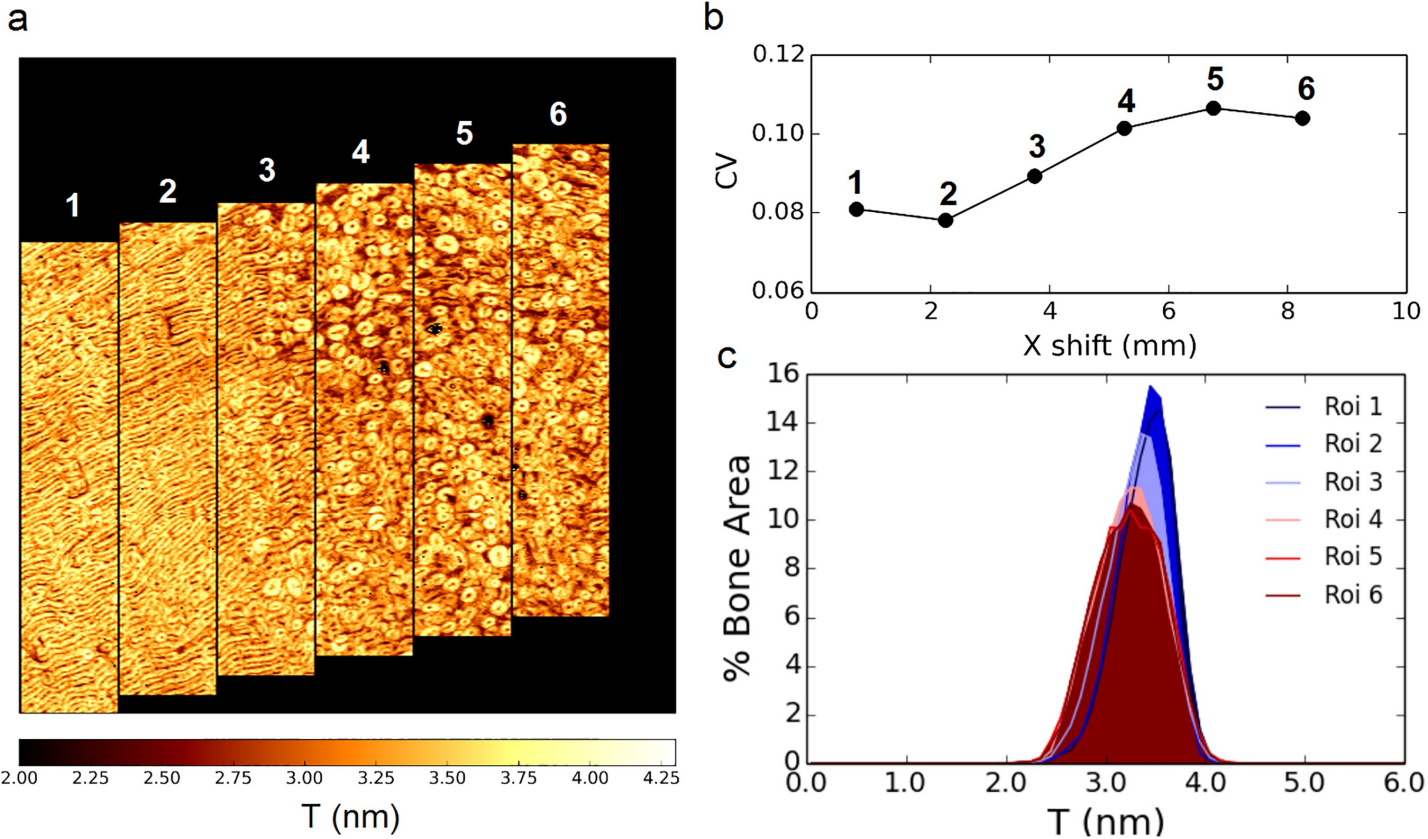
Intra-sample variability. (a) The qsSAXSI images of *T* (nm) shown in Fig 3D is divided into 6 regions of interest (roi) of width = 1.5 mm (scan width used for the heated samples). The vertical shift was chosen to ensure that the rois remain approximately centered with respect to the cortical shell. Corresponding (b) coefficient of variation and (c) image histograms. Scale bar: 1,5 mm.

In order to test the impact of the observed heterogeneity on a possible mismatch in sample alignment while setting up the subsequent scanning-SAXS measurements for the heated samples, the intra-sample variation was analyzed in the posterior reference section by subdividing the qsSAXSI image in adjacent regions of interest (roi) using the same width as this used for the scans of the heated samples to have statistically comparable data populations (Fig 5A). Qualitatively, it can be seen that the coefficient of variation (Fig 5B) increases between the fibrolamellar regions (roi 1–2) and the osteonal bone (roi 4–6).

To quantify this trend, the histograms of the images were calculated. The result is shown in Fig 5C where the shaded histograms represent the *T* distribution in percentage of the total bone area, i.e. disregarding the parts of the images corresponding to Haversian canals, osteocyte lacunae or other voids which appear in black in Figs 3D and 5A. A statistical analysis of the values in the different regions (~ 18000 points) reveals significant differences between all regions (p < 10^−6^) except between those in the osteonal bone (see S8 Table). However, the maximum differences found between the fibrolamellar and osteonal bone (roi 1–4, 1–5, 1–6) was estimated to Δ*T* = 0.18 nm and the difference between roi 1–2 in fibrolamellar bone was estimated to Δ*T* = 0.04 nm which is below the estimated resolution limit of 0.05 nm.

**Fig 6:**
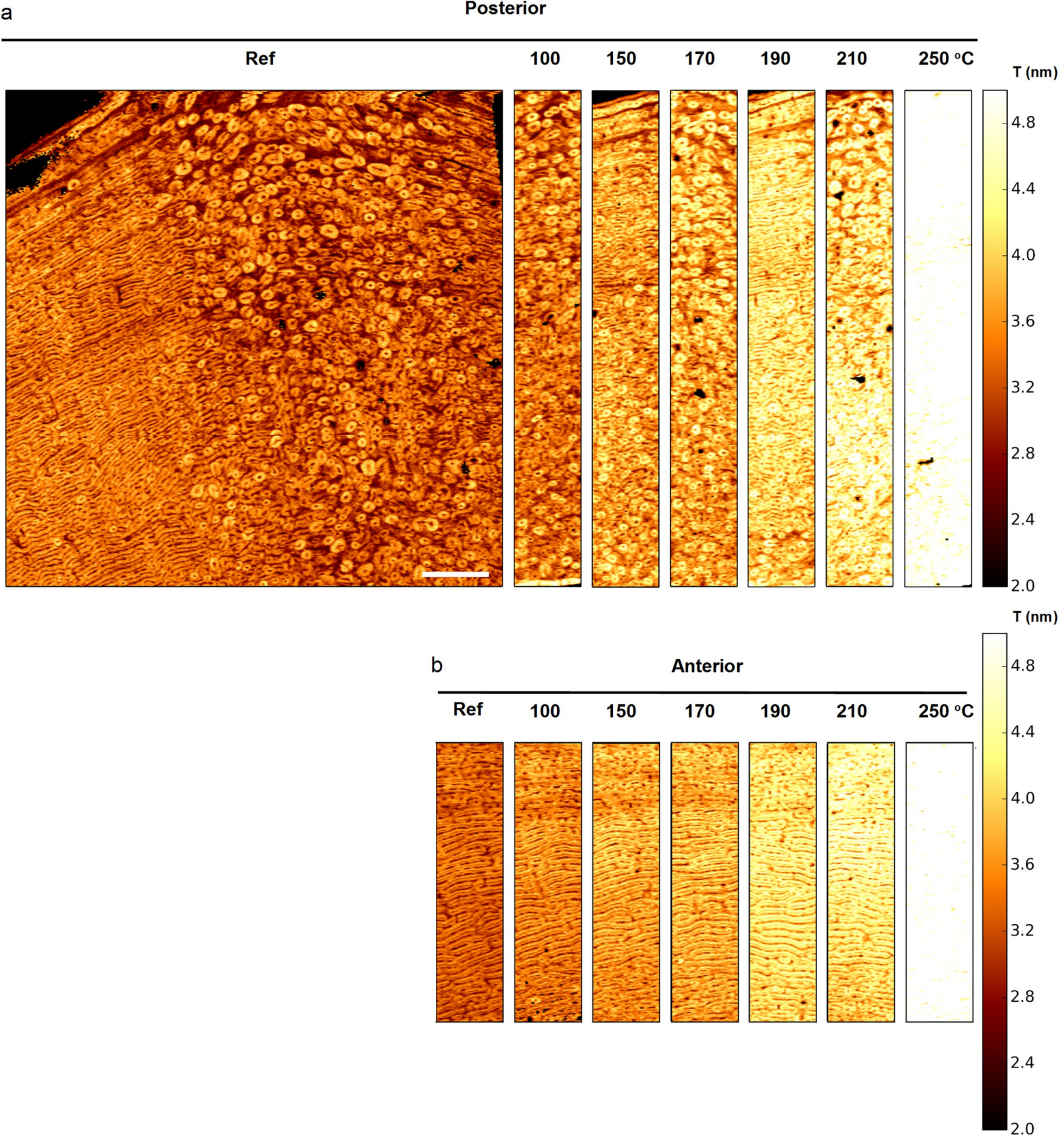
Mineral nanocrystal size at the tissue level. qsSAXSI images of *T* (nm) as a function of heating temperature for (a) the posterior and (b) the anterior sections. Due to sample damage during mounting for two of the anterior heated samples, only the centermost intra-cortical region is shown (see S3 Fig). The images are displayed on the same color and size scale for comparison. Scale bar: 1,5 mm.

A comparison between the qsSAXSI images of *T* obtained for all heated samples is shown in Fig 6. A clear increase of *T* as a function of temperature is observed in the form of an overall increase in brightness which is particularly important between 210°C and 250°C. Due to differences in sample positioning during the measurement and changes between adjacent sections, the samples could not be site-matched. Therefore, the images were analyzed using their histogram distributions.

**Fig 7:**
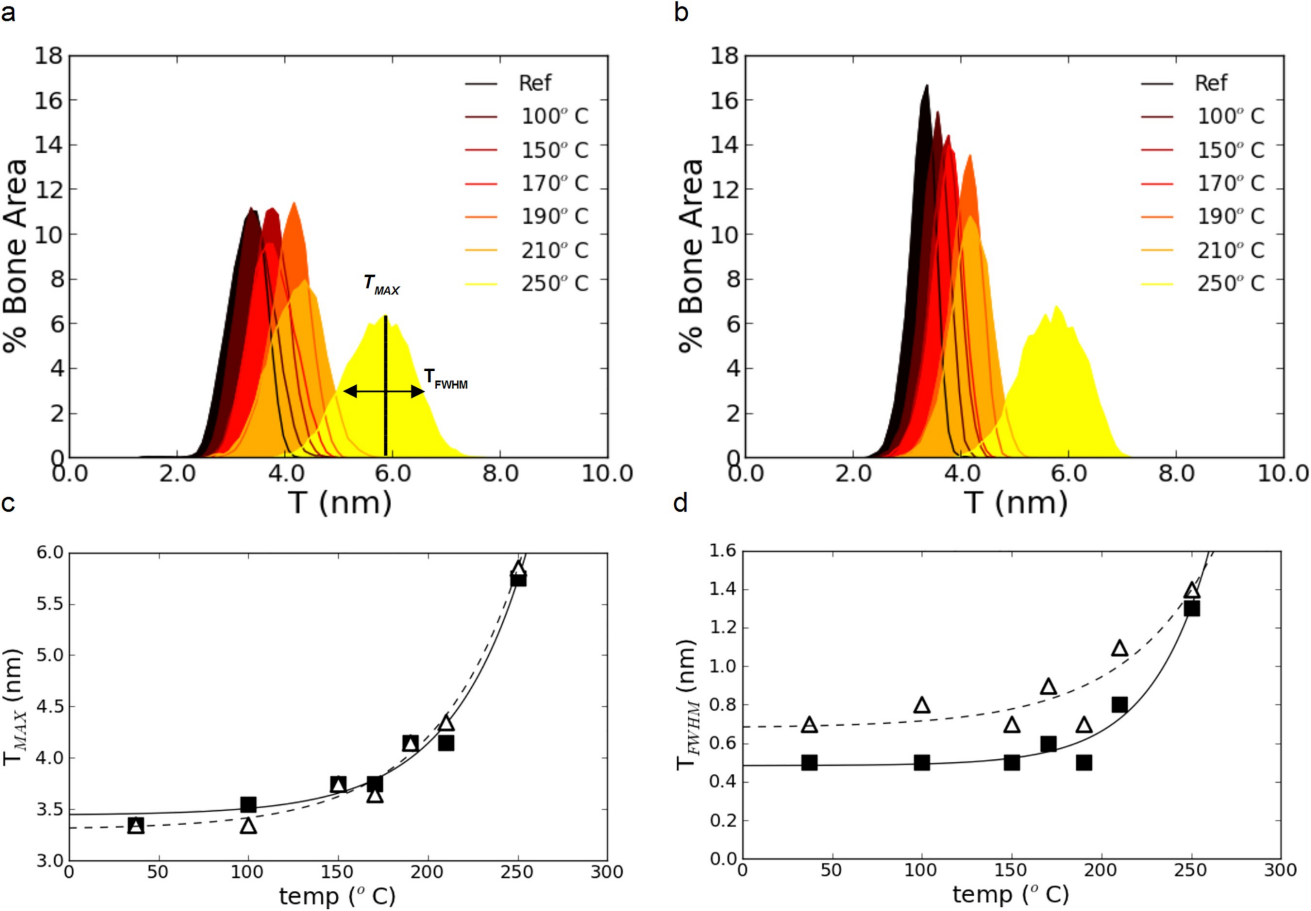
Quantification of the nanocrystal thickening. Histograms of the images of *T* as a function of temperature in (a) the posterior and (b) anterior regions. The position of the maximum (*T*_*MAX*_) and of the FWHM (*T*_*FWHM*_) obtained by fitting the distributions with a gaussian function are shown as a function of temperature in (c) and (d), respectively. The values for the posterior sections (open triangles) and the anterior one (black squares) reveal an exponential increase indicated by a dashed and a solid line.

The *T* distributions appear relatively Gaussian in shape (Fig 7). The position of the maximum of a given histogram can therefore be viewed as the average *T* value, while the full-width at half maximum (FWHM), provides an indication on the dispersion about the mean value.

A clear trend can be observed in the form of a shift towards higher values of *T* with increasing temperature which is correlated with a decrease of the maxima and a broadening of the histograms. Similar observations can be made for the *T* distributions of the anterior sections in Fig 7B. The physical interpretation of this trend is a general increase in particle thickness and in size distribution. Interestingly, the shift in peak position between 210°C and 250°C is approximately twice this measured between 37°C (reference) and 210°C which clearly suggests a non-linear trend. To quantify those changes, the histograms were fitted with a Gaussian function to extract the position of the maximum (*T*_*MAX*_) and the FWHM (*T*_*FWHM*_) as indicated in Fig 7A. Those parameters can be related to the average value and the variance of the *T* distribution. The results of the calculations are shown in Fig 7C and 7D. Both *T*_*MAX*_ and *T*_*FWHM*_ rise exponentially with increasing temperatures. The corresponding Spearman correlation coefficient is ρ = 0.98 for *T*_*MAX*_ anterior (p < 10^−3^), ρ = 0.95 for *T*_*MAX*_ posterior (p < 10^−3^), ρ = 0.81 for *T*_*FWHM*_ anterior (p = 0.03) and ρ = 0.70 for *T*_*FWHM*_ posterior (p = 0.08), emphasizing the strong correlation between particle size and temperature. Furthermore, all *T*_*MAX*_ values are significantly higher than those of the references as compared to the intra-sample variation estimated in the posterior reference sample (*ΔT*_*MAX*_ *>* 0.18 nm) except for the anterior group where not significant difference was observed between ambient and 100°C. However, a distinction can be made between the results obtained for the anterior (black squares) and posterior (open triangles) sections in *T*_*FWHM*_ which is not observed in *T*_*MAX*_. This suggests that the increase in average particle size (*T*_*MAX*_) is similar for the anterior and posterior samples but that there is a greater dispersion (variance) in particle size distribution (*T*_*FWHM*_) between the two anatomical quadrants.

### Microstructural interpretation of the qsSAXSI images

**Fig 8:**
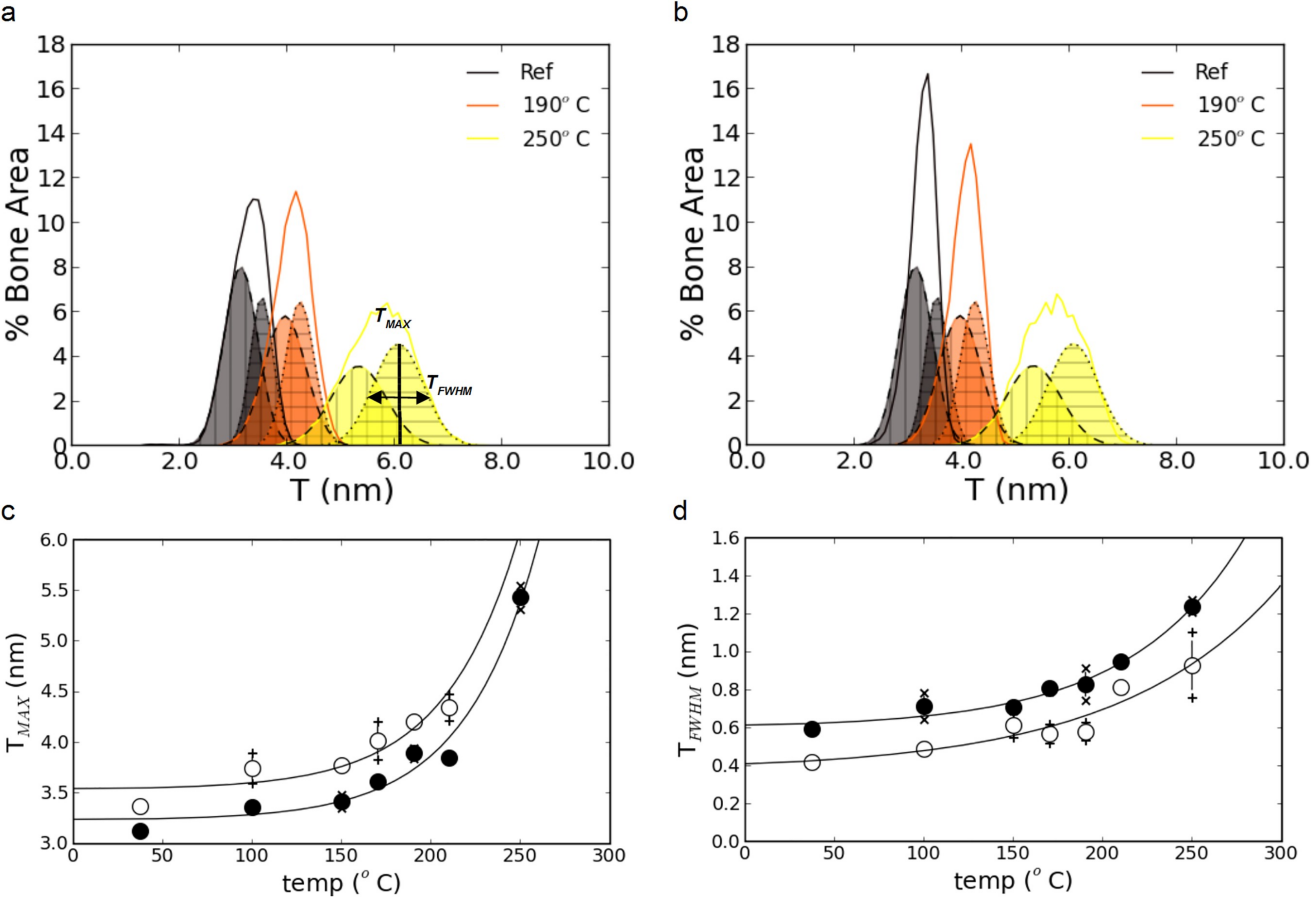
Modeling heat changes assuming two distinct populations of nanoparticles size. The fit of the *T* distributions shown in Fig 7 (solid linnes) using two Gaussian functions to account for the distinct particle populations is shown by dashed and dotted lines in (a) and (b). For clarity, only the histograms of the reference samples and those heated at 190°C and 250°C, which are well separated, are shown. The corresponding values of *T*_*MAX*_ and *T*_*FWHM*_ are shown in (c) and (d) where the open circles represent the gaussian fit with higher *T* values (horizontal hatching in (a) and (b)) and the closed circles represent the gaussian fit with lower *T* values (vertical hathing in (a) and (b)). For each particle population, the crosses indicate the results obtained in the anterior or posterior sections while the circles represent the average values.

In order to understand the apparent differences in the evolution of *T*_*MAX*_ and *T*_*FWHM*_ for the anterior and posterior samples, the following points need to be considered. A closer examination of the histograms in Fig 7A and 7B reveals a moderate negative skew which is observed in all curves. This is a typical signature for the presence of two (possibly more) distinct, albeit overlapping, particle populations. This hypothesis is a direct consequence of the observations based on the images in Fig 3D and Fig 4, highlighting the differences between bone tissue exhibiting high *T* values (indicated by stars in Fig 4B and 4C) and lower ones (arrows in Fig 4B and 4C). Consequently, the *T* distributions were fitted using Gaussian functions represented by dashed and dotted lines for the anterior (Fig 8A) and the posterior (Fig 8B) sections.

Two Gaussian functions were sufficient to obtain a high fitting quality with R^2^ values > 0.995, thus strengthening our hypothesis of two distinct nanoparticle populations. The values of *T*_*MAX*_ and *T*_*FWHM*_ derived from the fitting procedure with two Gaussians are shown in Fig 8C and 8D respectively. The fact that the crosses are very close to their average values for each temperature shows that nanoparticles with lower thickness are comparable in the anterior and posterior sections and the same holds for the thicker ones. Furthermore, the exponential trends for *T*_*MAX*_ and *T*_*FWHM*_ are extremely close (Spearman ρ = 0.96 for *T*_*MAX*_ upper (p < 10^−3^), ρ = 0.99 for *T*_*MAX*_ posterior (p < 10^−3^), ρ = 0.96 for *T*_*FWHM*_ anterior (p < 10^−3^) and ρ = 0.89 for *T*_*FWHM*_ posterior (p < 0.01)), the only significant difference being a relative shift between the curves obtained for the two populations.

### Analysis of the thermally activated process

**Fig 9:**
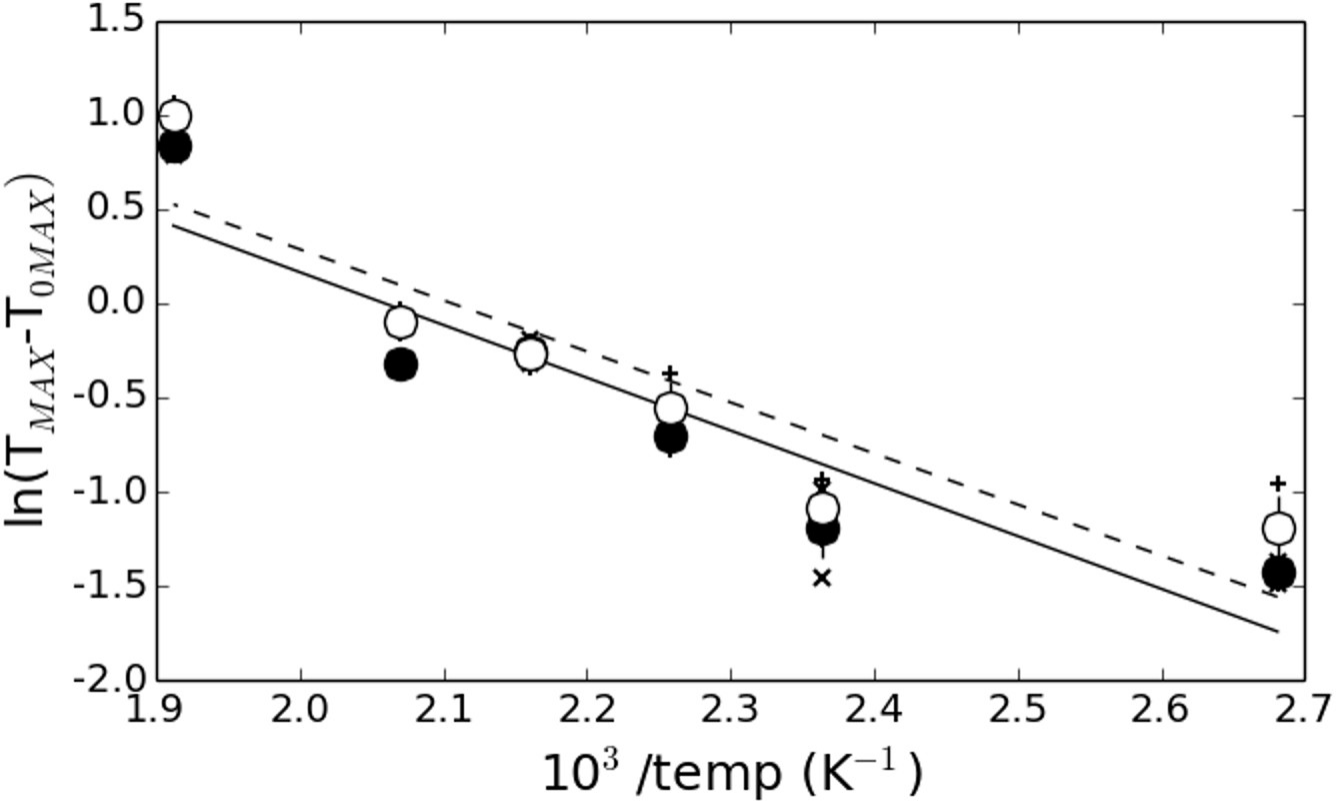
Estimation of the activation energy of the growth process. Arrhenius plot of the *T*_*MAX*_ data shown in the Fig 8A using the same conventions. The solid and dashed lines indicate the result of the linear regression.

The positive correlation observed for *T*_*MAX*_ and *T*_*FWHM*_ in the posterior and anterior sections for the osteons or the interstitial tissue suggests a similar nanoparticle growth process. Most growth processes can be described by an Arrhenius law of the type:

$$ln(T_{MAX}-T_{0MAX})=\frac{1}{n}ln(t-t_{0})-\frac{E_{a}}{nRT^{K}}$$ (4)

where *T*_*0MAX*_ is the average particle size at room temperature (25°C), *t*–*t*_0_ is the heating time, *T*^*K*^ is the temperature, *R* is the gas constant (8.31 J.mol^-1^.K^-1^), *E*_*a*_ is the activation energy and *n* is a dimensionless coefficient which is generally derived from the isothermal measurements. Due to the lack of isothermal data in this study, this latter parameter is unknown. However, this is also the case for many similar experiments and a common approximation is *n* = 1 [62]. The data were therefore plotted in the form of ln(*T*_*MAX*_−*T*_*0MAX*_) vs. 10^3^/T^K^ and fitted using a linear regression. The intercept allows determining the value of *n* and the slope provides an estimation of *Ea*. The result is shown in Fig 9 using the same conventions as Fig 8C and 8D.

The dashed line represents the result of the linear fit of the average value of *T*_*MAX*_ for the higher *T* distributions (open circles) of the anterior and posterior sections (vertical crosses) while the solid line represents the same information for the lower *T* distributions (closed circles with diagonal crosses). The calculated activation energy was *E*_*a*_ = 33.1 KJ.mol^-1^ (*n* = 1.42) and *Ea* = 32.3 KJ.mol^-1^ (*n* = 1.44) respectively. These values are consistent with results from the literature on solid state diffusion processes but should still be considered with caution since they depend on the estimation of *n*. Nevertheless, they are relatively close, suggesting that the dynamical processes are very similar in the two types of tissue independently of anatomical quadrant location.

### Characteristics of mineral nanoparticle organization

**Fig 10:**
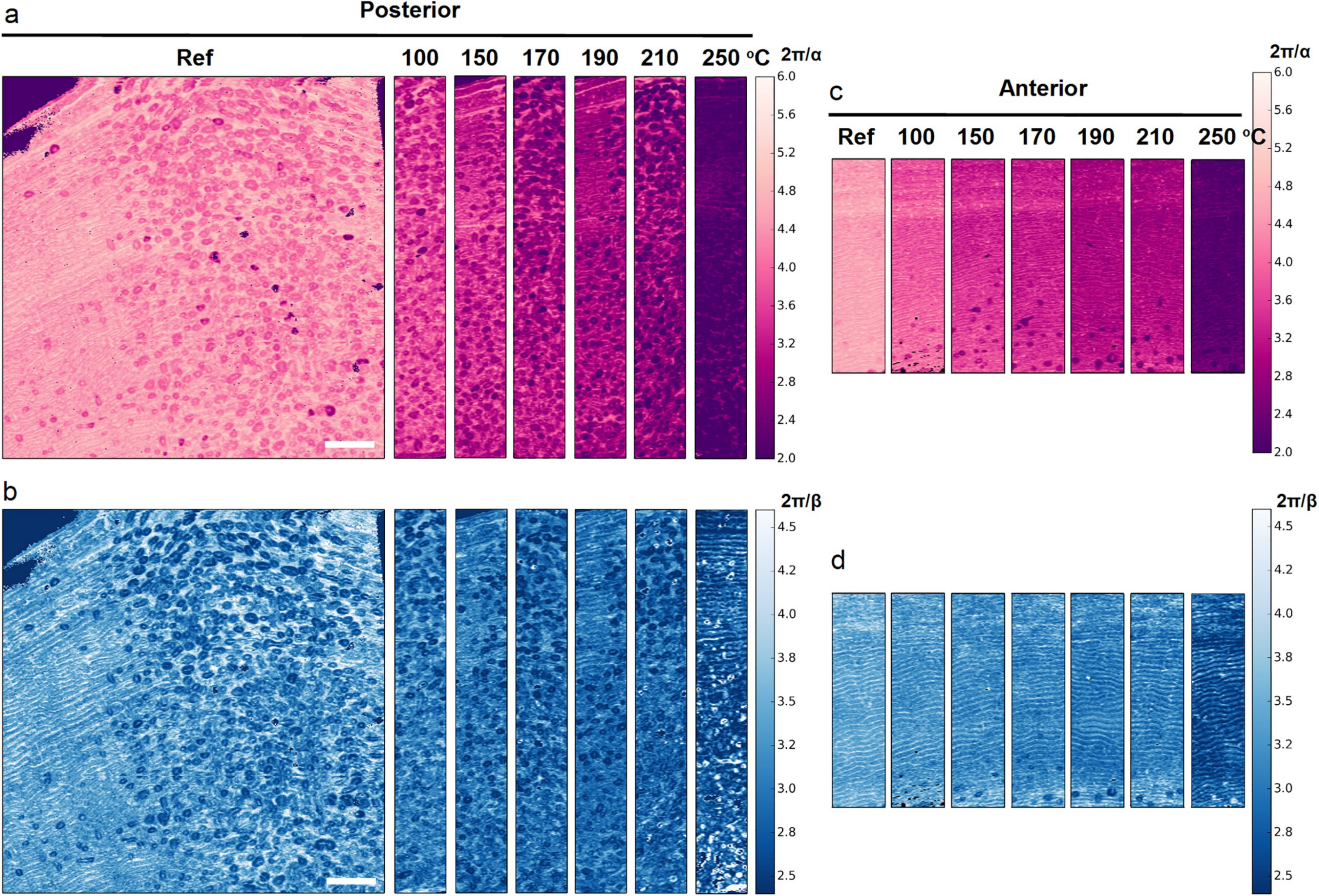
Mineral nanocrystal organization at the tissue level. qsSAXSI images of 2π/*α* vs T.2π/*β* (nm) as a function of heating temperature for (a,b) the posterior and (c,d) the anterior sections. The images are displayed on the same color and size scale for comparison. Scale bar: 1,5 mm.

In light of the analysis of mineral nanoparticle size, it is also interesting to consider the qsSAXSI images of 2π/*α* (Fig 10A and 10C) and 2π/*β* (Fig 10B and 10D) which describe the degree of regularity in the nanoparticle organization based on the stack of cards model. In both cases, the distinction between osteons and interstitial tissue in osteonal bone is clearly apparent in the reference sample, with lower values of 2π/*α* and 2π/*β* for osteonal bone than interstitial tissue. The spatial distribution of both parameters was found to be well correlated with the polarized light microscopy image of the reference posterior sample (S4 and S5 Figs). Upon heating, a progressive change in both parameters is observed, particularly between 210°C and 250°C. However, the observed decrease in 2π/*α* appears to be less than this of 2π/*β*, on average.

**Fig 11:**
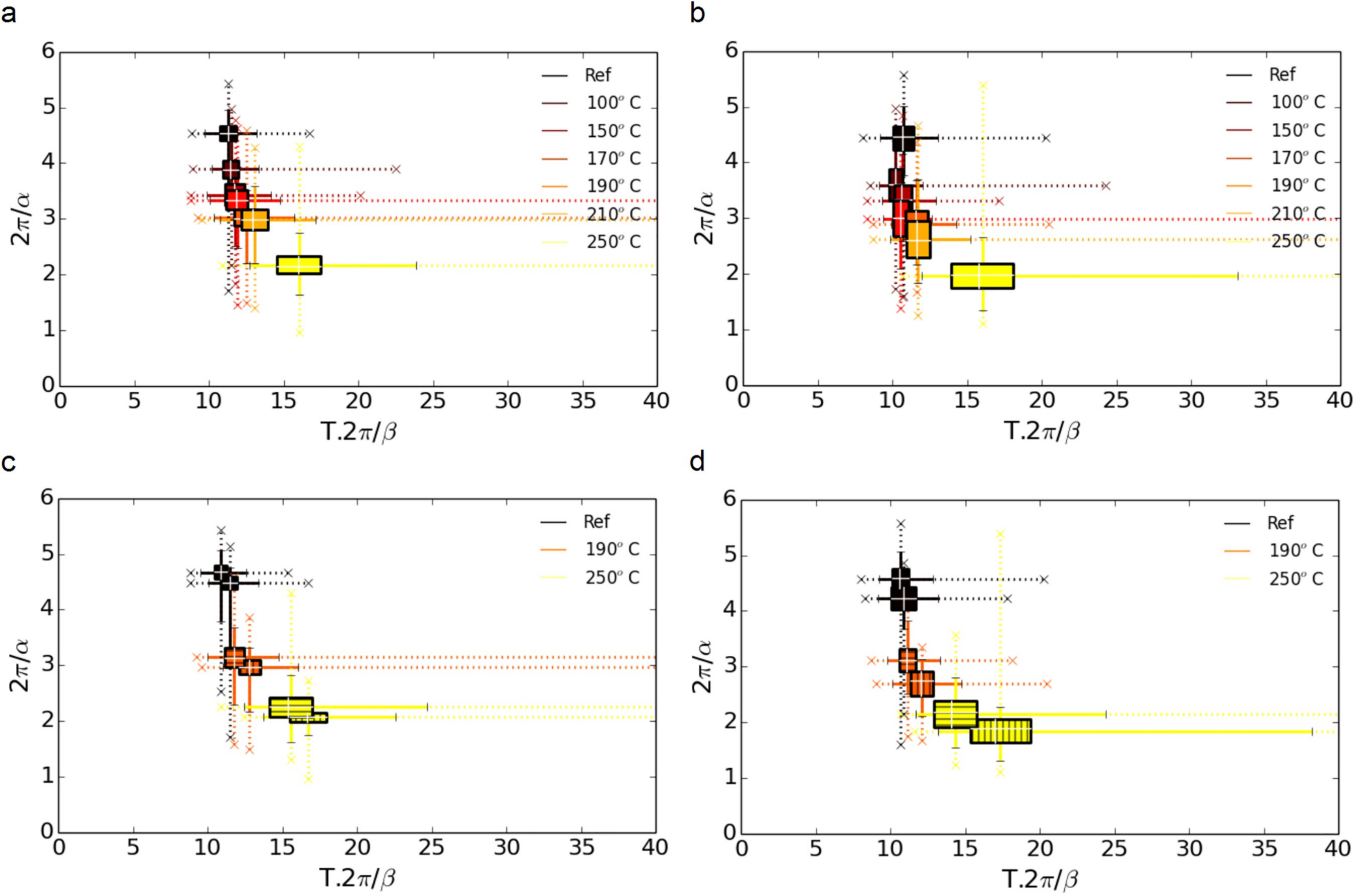
Perturbation in the mineral nanocrystal organization. Plot of 2π/*α* vs T.2π/*β*, which characterizes the degree of lateral ordering in bone using the Stack-of-cards model, in (a) the anterior and (b) the posterior regions as a function of temperature. The rectangles represent the inter-quartile range (containing 50% of the data) of the values shown in Fig 10. The solid lines, positioned at the median values, show the limits within which 95% of the data can be found. The remaining 5% fall within the range indicated by dotted lines with lower and upper limits indicated by crosses for clarity. The statistics for the two populations of particles, are shown in (c) for the anterior region and (d) for the anterior one using the same conventions than Fig 8, i.e. a horizontal hatching for higher *T* values and vertical hatching for the lower *T* values. For clarity, only the values obtained for the reference samples and those heated at 190°C and 250°C are shown in (b) and (d).

To quantify those trends, the statistical distribution of the two parameters is shown in Fig 11. This representation was previously used to analyze pathological modifications in human mineral nanoscale organization [43]. Both data sets from the anterior (Fig 11A) and the posterior (Fig 11B) regions reveal a gradual shift to smaller 2π/*α* values, indicating a loss of regularity in the nanoparticle stacking and to higher interparticle distance *T*.2π/*β*.

Also, there is a progressive increase in the dispersion of the values as indicated by the increased width of the interquartile range (width of the box), interdecile range (extent of the solid lines) and extreme values (extent of the dotted lines), particularly along the horizontal axis. Although the trends are similar in both anatomical quadrants, differences can be observed between the two in Fig 11A and 11B. Thus, a mask image was defined based on the thresholded *T* images at all temperatures to retrieve the corresponding values of 2π/*α* and 2π/*β* for the populations with high and low *T* in the anterior (Fig 11C) and posterior quadrants (Fig 11D). In addition to thickness, the two nanoparticle populations were, thus, also found to be distinct in terms of organization with slightly higher interparticle distances (*T*.2π/*β*) and lower packing regularity (2π/*α*) for the smaller nanoparticles. However, the general trend of a progressive loss of organization is similar between the two particle populations independent of the anatomical location which is most likely due to the collagen partial denaturation.

## Discussion

The previous analysis points to significant nanoscale structural modifications of bone upon heating, even at temperatures < 250°C. The modifications observed by raman spectroscopy and TEM fully support the conclusions from previous studies of a progressive evaporation of tightly bound water and collagen denaturation [2,9–10,18,54]. In addition, our Raman, TEM and SAXS analysis also indicate a continuous, non-monotonic mineral nanoparticle growth. At first sight this seems to be contradictory to earlier studies conducted on artificially heated bone in the archaeological context which concluded the absence of changes in the X-ray diffraction (XRD) signal of the mineral phase at temperatures lower than 300°C [19–21]. In particular, mineral particle size estimated by peak broadening of the XRD radial profiles was found to be essentially constant. Similar conclusions were reached in a recent study combining SAXS and XRD *in situ* on dentin, although subtle structural changes can be observed below 300°C that were not discussed by the authors [63]. While our Raman measurements confirm a relative stability in the mineral crystal chemistry up to 210°C, the SAXS result clearly show a significant increase in *T*. However, other studies show that the mineral nanocrystal size estimated by XRD line broadening techniques do not accurately reflect the dimensions measured by TEM [64]. SAXS, was found to be accurate in this respect [39–41]. This can be rationalized by the fact that the SAXS signal is sensitive to the electron density contrast between the organic and mineral phases while XRD is sensitive to the crystalline fraction of the mineral nanoparticles. If the material re-deposited upon heat treatment is poorly ordered on the atomic scale, then the crystalline unit calculated by XRD may be smaller than the overall particle shape observed by SAXS. Structural modifications of the collagen resulting from heating could induce a bias in the particle size estimation by SAXS. However, 1) our spectroscopy results show that the conformational changes are limited within the experimental temperature range, as reported in early X-ray studies [52] and 2) the nanoparticle thickness measured by TEM analysis are in good agreement with the SAXS results. Clearly, although we cannot exclude an effect due to structural changes of the organic matrix, this will only have a minor influence on the calculation of *T* (following Eq 1, a decrease as high as 10% in *Φ* would only result in a 4% change in *T*). Therefore, the increase in *T* can unambiguously be attributed to a change in mineral nanoparticles dimensions.

As a consequence of the modifications of the organic phase, the mineral nanoparticles were found to be highly disorganized at temperatures > 170°C as observed by TEM (Fig 2), although sample preparation artifacts resulting from the softening of the organic matrix cannot be excluded. However, the thin, elongated platelet shape of the mineral phase remained essentially conserved. Two aspects of the SAXS measurements were found to be essential for the nanoscale characterization of bone. 1) Due to the highly localized volume imaged in TEM (~ 1 μm^2^ field of view for 1.28 nm^2^ spatial resolution), the relevance of this information is not really clear for a material as heterogeneous as bone in which the microstructural features are typically 100–200 μm in length. To overcome this limited field of view, we took advantage of the very high X-ray synchrotron flux which allows exploring large sample regions by scanning [47]. The information derived from the analysis is therefore statistically significant, in a biological sense, which is not the case with TEM. On the other hand, a phenomenological understanding is difficult to obtain from the sole SAXS measurements, such that both information are required. 2) By tuning the X-ray beam to a diameter to 15 × 20 μm^2^, which is smaller than the size of the microstructural features, we were able to evidence differences in mineral nanoscale size and organization between distinct tissue types. Due to the intricate organization between the collagen structure and the mineral phase, the changes in organization are most likely due to collagen disorganization, as also described in a previous medical study [43]. Added to this the ability to scan large sample regions (> 1 cm^2^ for the reference sample of the posterior quadrant in Fig 3) [65], qsSAXSI provides a unique possibility to put the nanoscale mineral characteristics in perspective with sample histology as highlighted by the combination of qsSAXSI and PL images in Fig 4. This should be seen a true (direct) multiscale analysis of bone ultrastructure.

From the results obtained by qsSAXSI, an important consequence follows: the interpretation of the nanoscale analysis of the mineral phase may be biased by the microstructural heterogeneity at the tissue level. This is illustrated by the differences between Fig 7 and Fig 8. When considering the average particle thickness, *T*_*MAX*_, no significant difference was found between the samples prepared from the anterior and posterior sections (Fig 7C). However, there is a significant difference between the two quadrants in the variance of the particle thickness, *T*_*FWHM*_ (Fig 7D). Therefore, the mineral ultrastructure seems statistically different in the two anatomical locations. The analysis based on the histology clearly shows that this is not the case: the nanoscale properties differ between osteons and interstitial tissue in osteonal bone and between lamellar and fibrous tissue in fibrolamellar bone by an offset in *T*_*MAX*_ (Fig 8C) and *T*_*FWHM*_ (Fig 8D) but they are strictly equivalent in the anterior and posterior regions. The difference observed in Fig 7 can only be explained by the relative amount of tissue of both types which differs in the two anatomical quadrants and is, therefore, site-dependent. Hence, if different coexisting tissues have distinct nanoscale characteristics, the balance between their volume fractions may influence the macroscopically averaged properties of bone.

From a biological point of view, it is now well established that the primary and secondary osteons are formed during modeling and remodeling phases and are, therefore, more recent than interstitial tissue. Similarly, the fibrous tissue is known to form prior to lamellar bone. The most recent tissues are generally associated with a lower degree of mineralization and mineral maturity [66]. Our observations tend to indicate that, although the growth process is most likely identical for the different types of structures, the initial state of mineralization defines the extent of the particle growth upon heating. Should our observation be confirmed in future studies, the absolute values of *T*_*MAX*_ and *T*_*FWHM*_ could be used in forensic and archaeological applications as an indicator of the temperature of heating, while the relative shift in *T*_*MAX*_ and *T*_*FWHM*_ between tissue types encountered in a sample could provide clues to the age of the tissue and/or the duration of heating. This conclusion requires some caution since the experiment was essentially limited to a single heating time and, due to the necessary adaptation of the samples to the specific requirements of each analytical modality, the measurements were not all standardized to the same measuring volumes. Furthermore, the samples used in this study were far from their native state. The role of water on such heat-induced structural changes, in particular, needs to be taken into account. While dehydration is a requirement for TEM measurements, both SAXS and Raman measurements can readily be performed on fresh bone samples [45,67]. Moreover, in this study, the micro-Raman analysis was essentially limited to the heating effects on the organic matrix in order to assess such impact on the qsSAXSI analysis. However the potential of Raman spectroscopy used in a scanning imaging mode for compositional analysis has been demonstrated by other authors [68–69] and could be used advantageously to further investigate the effect of heat changes at the molecular scale using a similar approach as this proposed for qsSAXSI.

## Conclusion

Using a combination of Raman microspectroscopy, TEM and qsSAXSI, we analyzed a bovine cortical bone model showing that heating at temperatures < 250°C induces a strong disorganization and increase in thickness of the mineral nanoparticles that were not detected in previous XRD studies. Furthermore, the measurements performed with synchotron X-ray microbeams in the 10–20 μm size range revealed important differences in the nanoscale characteristics of different bone tissue types, reflecting the structure of fibrolamellar and osteonal bone. Using a microstructural based segmentation of the data, we found that the statistical differences between samples prepared from the posterior and anterior regions could be explained by the relative amount of tissue type, but that the nanoscale properties of the individual tissues were identical in both anatomical locations. We, thus, show that nanoscale investigations of materials which are heterogeneous at higher length scales should be analyzed on the basis of the heterogeneity distribution. In the particular case of biological tissues, this entails a histological interpretation or, in a broader sense, this requires putting the nanostructural characteristics in perspective with the microstructural heterogeneity.

## Supporting information

S1 Fig Raman spectroscopy data. (a) Raw average Raman spectra (before background subtraction) as a function of temperature. Note the increasing background and the total loss of signal at 250°C. (b-e) Background subtracted Raman spectra for the 10 measurements collected at room temperature (b), 150°C (c), 190°C (d) and 210°C (e). The spectra show a variable amount of residual background. No significant differences between the spectra collected in the different tissue type could be found. Hence, the spectra were pooled for analysis.
(PDF)

S2 Fig Polarized light microscopy image of the reference sample in the posterior region. (a) Raw PLM image. The region scanned by qsSAXSI is indicated by the red rectangle. (b) Enlarged view of the region indicated by a white rectangle in (a) showing the transition between osteonal bone (left) and fibrolamellar bone (right). Scale bars: 1,5 mm in (a) and 0,5 mm in (b).
(PDF)

S3 Fig Full qsSAXSI images of the reference and heated samples. The samples heated at 100°C and 150°C were damaged during mounting and could not be fully scanned. In order to provide comparable areas, only the data contained within the dashed blue lines (shown in Fig 6) were considered for subsequent analysis.
(PDF)

S4 Fig Site-matched correspondence between the tissue microstructure and tissue organization. Overlay of the qsSAXSI image of 2π/*α* (pink) and the polarized light microscopy image (green) of the reference sample in the posterior region. Note that lower values of 2π/*α*, indicating a reduced spatial extent of the regularity in nanoparticle packing, can be observed in osteonal bone. Scale bars: 1,5 mm.
(PDF)

S5 Fig Site-matched correspondence between the tissue microstructure and tissue organization. Overlay of the qsSAXSI image of 2π/*β* (blue) and the polarized light microscopy image of the reference sample in the posterior region. Note that the lower values of 2π/*β* indicating a closer nanoparticle packing correspond to the osteons and higher ones to interestitial tissue in the osteonal zone. Scale bars: 1,5 mm.
(PDF)

S1 Table Result of two sided Mann-Whitney statistical test for the ν1PO4 band. (p-values and estimated 90% confidence interval for the difference between two populations).
(PDF)

S2 Table Result of two sided Mann-Whitney statistical test for the ν1CO3 band. (PDF)

S3 Table Result of two sided Mann-Whitney statistical test for the νAmide I band. (PDF)

S4 Table Result of two sided Mann-Whitney statistical test for the νCH band. (PDF)

S5 Table Result of two sided Mann-Whitney statistical test for the ν1CO3/ ν1PO4 intensity ratio. (PDF)

S6 Table Result of two sided Mann-Whitney statistical test for the ν1PO4/ νCH intensity ratio. (PDF)

S7 Table Result of two sided Mann-Whitney statistical test for the particle sized derived by TEM. (PDF)

S8 Table Result of two sided Mann-Whitney statistical test for the particle sized derived by qsSAXSI. (PDF)
