# Supplementary material for: Nanoscale modifications in the early heating stages of bone are heterogeneous at the microstructural scale
Source: PLoS One. 2017 Apr 19;12(4):e0176179. doi: 10.1371/journal.pone.0176179 (PMC5397064; doi:10.1371/journal.pone.0176179)

S1 Fig. Raman spectroscopy data. (a) Raw average Raman spectra (before background subtraction) as a function of temperature. Note the increasing background and the total loss of signal at 250 ^{o}C. (b-e) Background subtracted Raman spectra for the 10 measurements collected at room temperature (b), 150 ^{o}C (c), 190 ^{o}C (d) and 210 ^{o}C (e). The spectra show a variable amount of residual background. No significant differences between the spectra collected in the different tissue type could be found. Hence, the spectra were pooled for analysis.

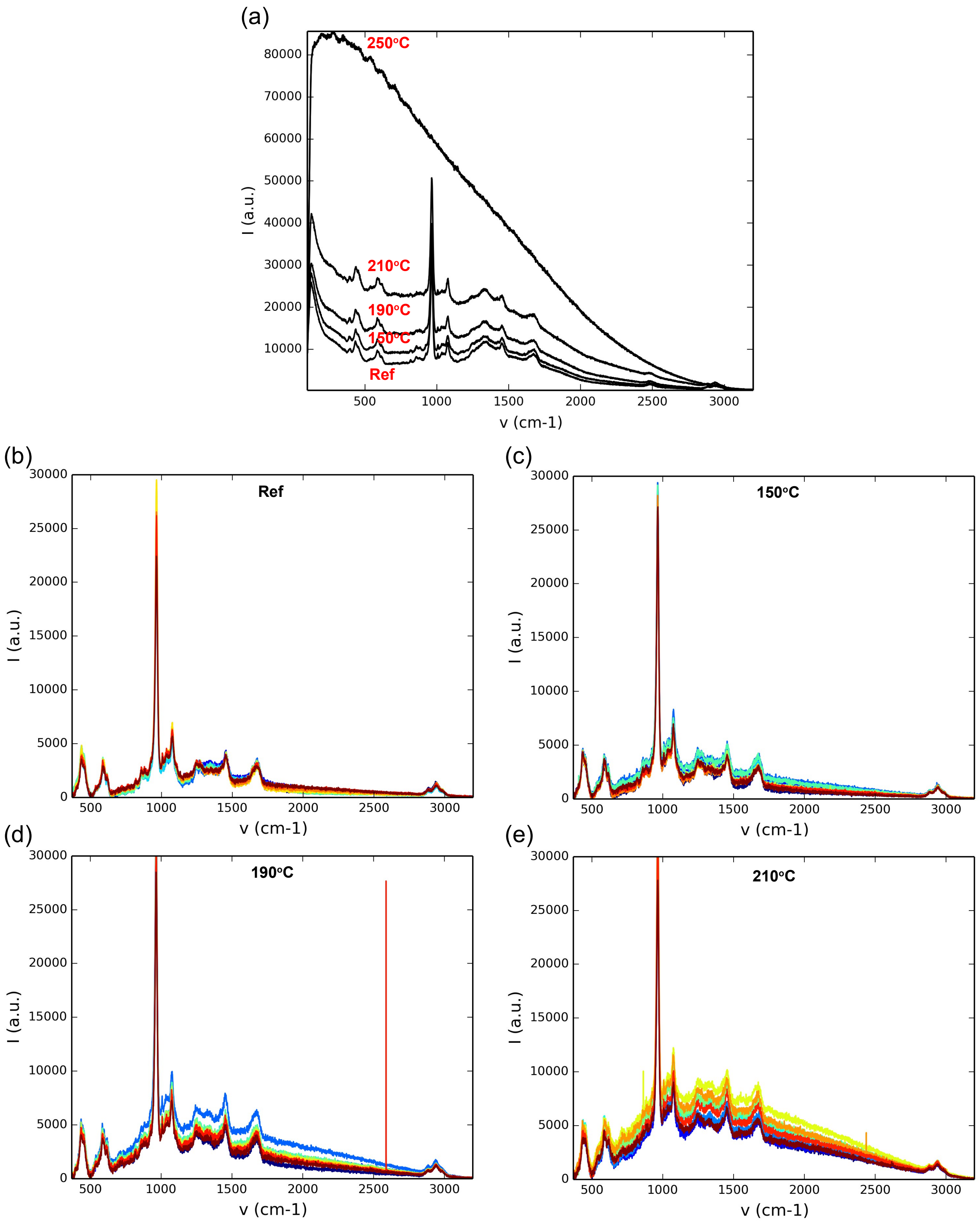

Supplement: S1 Fig — (a) Raw average Raman spectra (before background subtraction) as a function of temperature. Note the increasing background and the total loss of signal at 250°C. (b-e) Background subtracted Raman spectra for the 10 measurements collected at room temperature (b), 150°C (c), 190°C (d) and 210°C (e). The spectra show a variable amount of residual background. No significant differences between the spectra collected in the different tissue type could be found. Hence, the spectra were pooled for analysis. (PDF) [file pone.0176179.s001.pdf]
