# Supplementary material for: Nanoscale modifications in the early heating stages of bone are heterogeneous at the microstructural scale
Source: PLoS One. 2017 Apr 19;12(4):e0176179. doi: 10.1371/journal.pone.0176179 (PMC5397064; doi:10.1371/journal.pone.0176179)

S2 Fig. Polarized light microscopy image of the reference sample in the posterior region. (a) Raw PLM image. The region scanned by qsSAXSI is indicated by the red rectangle. (b) Enlarged view of the region indicated by a white rectangle in (a) showing the transition between osteonal bone (left) and fibrolamellar bone (right). Scale bars: 1,5 mm in (a) and 0,5 mm in (b).

a

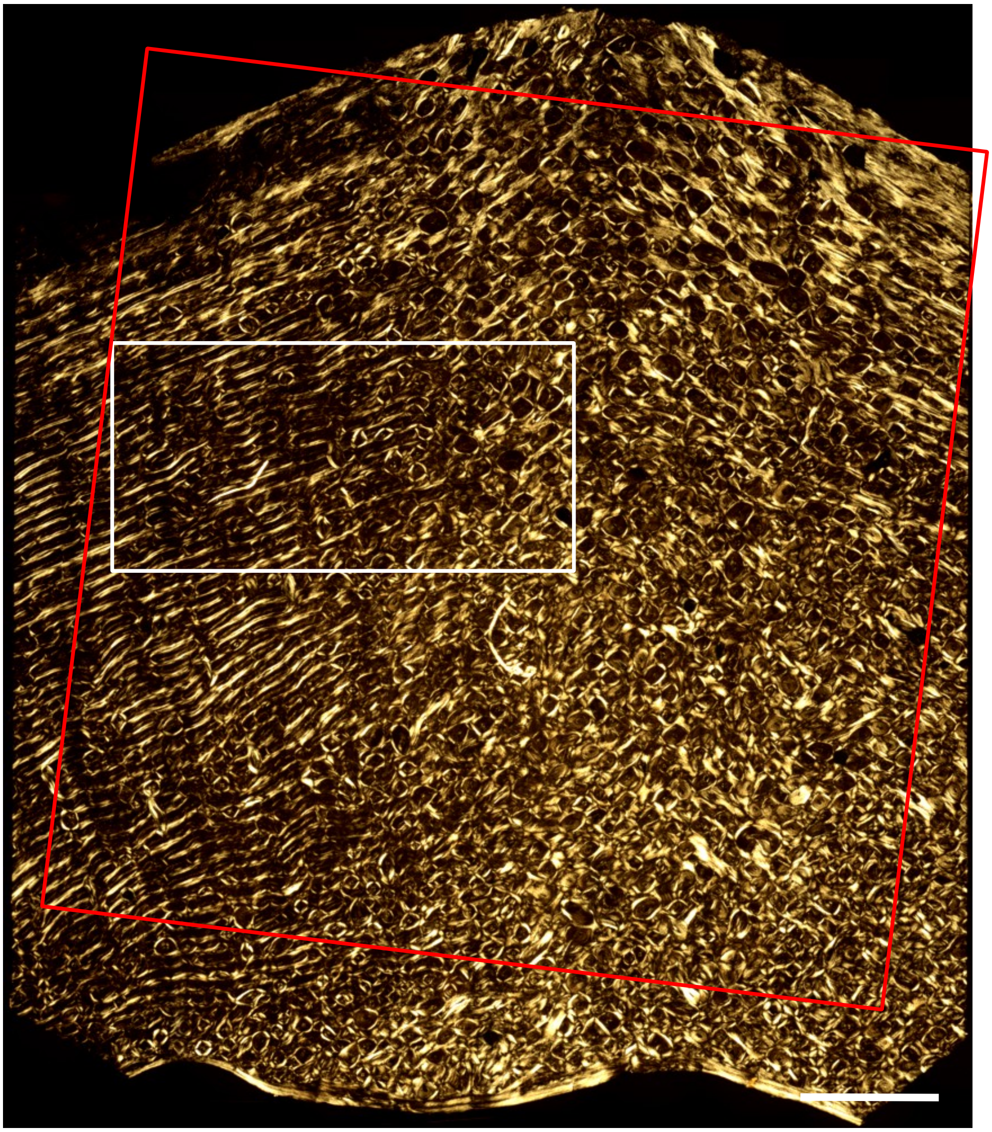

b

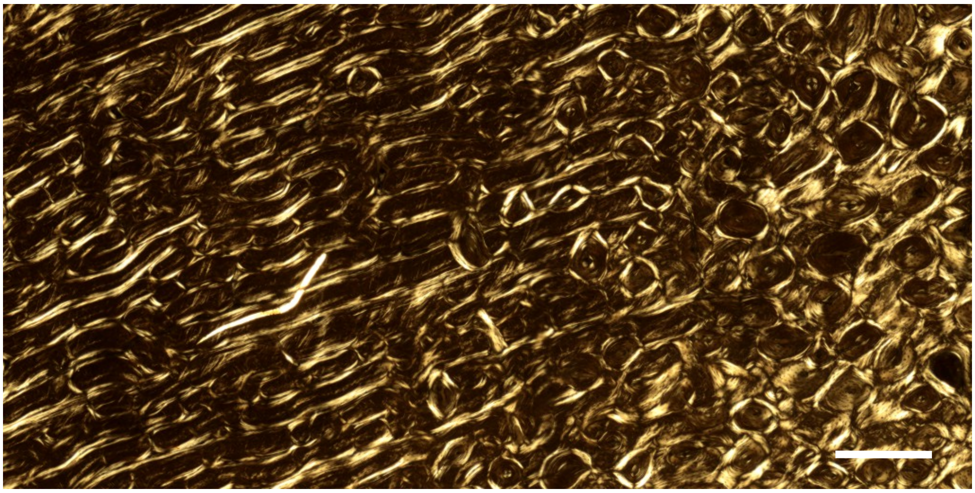

Supplement: S2 Fig — (a) Raw PLM image. The region scanned by qsSAXSI is indicated by the red rectangle. (b) Enlarged view of the region indicated by a white rectangle in (a) showing the transition between osteonal bone (left) and fibrolamellar bone (right). Scale bars: 1,5 mm in (a) and 0,5 mm in (b). (PDF) [file pone.0176179.s002.pdf]
