# Supplementary material for: Nanoscale modifications in the early heating stages of bone are heterogeneous at the microstructural scale
Source: PLoS One. 2017 Apr 19;12(4):e0176179. doi: 10.1371/journal.pone.0176179 (PMC5397064; doi:10.1371/journal.pone.0176179)

S3 Fig. Full qsSAXSI images of the reference and heated samples. The samples heated at 100 °C and 150 °C were damaged during mounting and could not be fully scanned. In order to provide comparable areas, only the data contained within the dashed blue lines (shown in Fig 6) were considered for subsequent analysis.

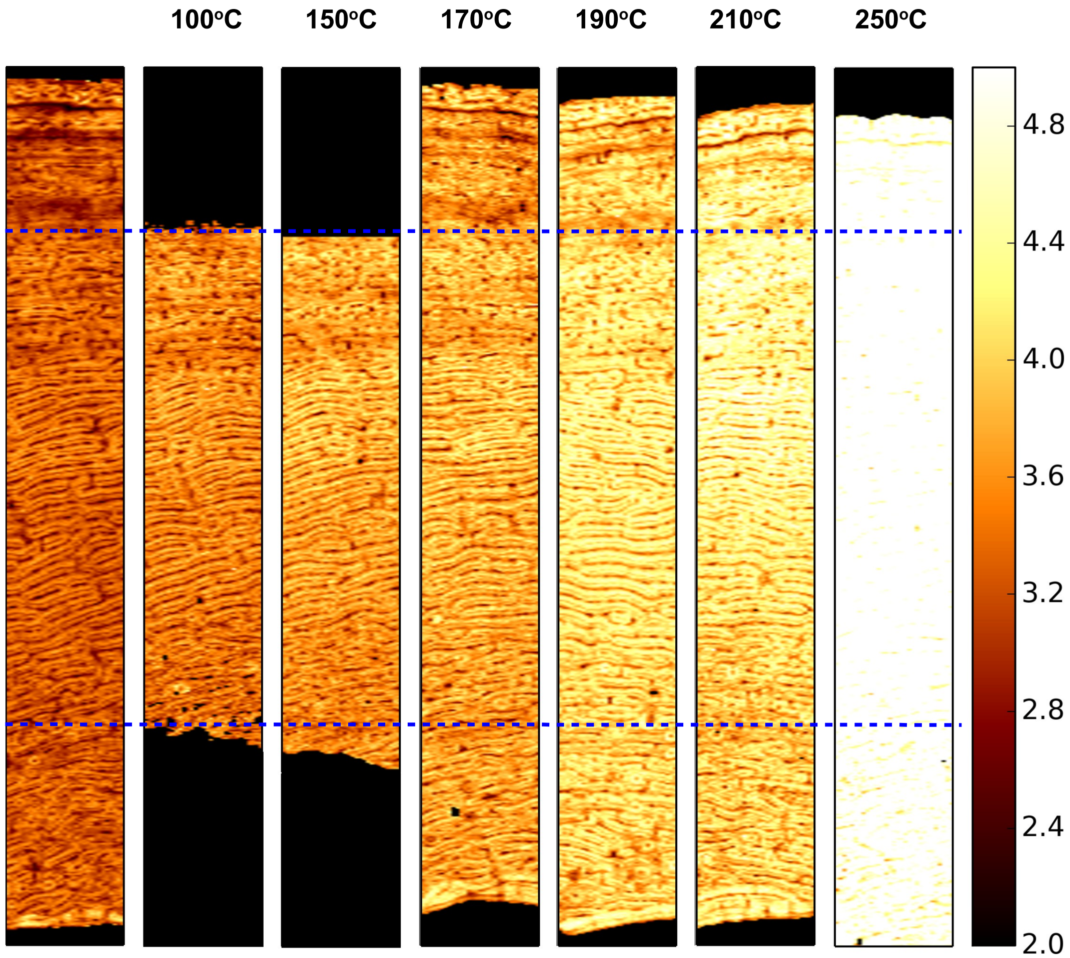

Supplement: S3 Fig — The samples heated at 100°C and 150°C were damaged during mounting and could not be fully scanned. In order to provide comparable areas, only the data contained within the dashed blue lines (shown in Fig 6) were considered for subsequent analysis. (PDF) [file pone.0176179.s003.pdf]
