# Supplementary material for: Nanoscale modifications in the early heating stages of bone are heterogeneous at the microstructural scale
Source: PLoS One. 2017 Apr 19;12(4):e0176179. doi: 10.1371/journal.pone.0176179 (PMC5397064; doi:10.1371/journal.pone.0176179)

S4 Fig. Site-matched correspondence between the tissue microstructure and tissue organization. Overlay of the qsSAXSI image of $2\pi/\alpha$ (pink) and the polarized light microscopy image (green) of the reference sample in the posterior region. Note that lower values of $2\pi/\alpha$, indicating a reduced spatial extent of the regularity in nanoparticle packing, can be observed in osteonal bone. Scale bars: 1,5 mm.

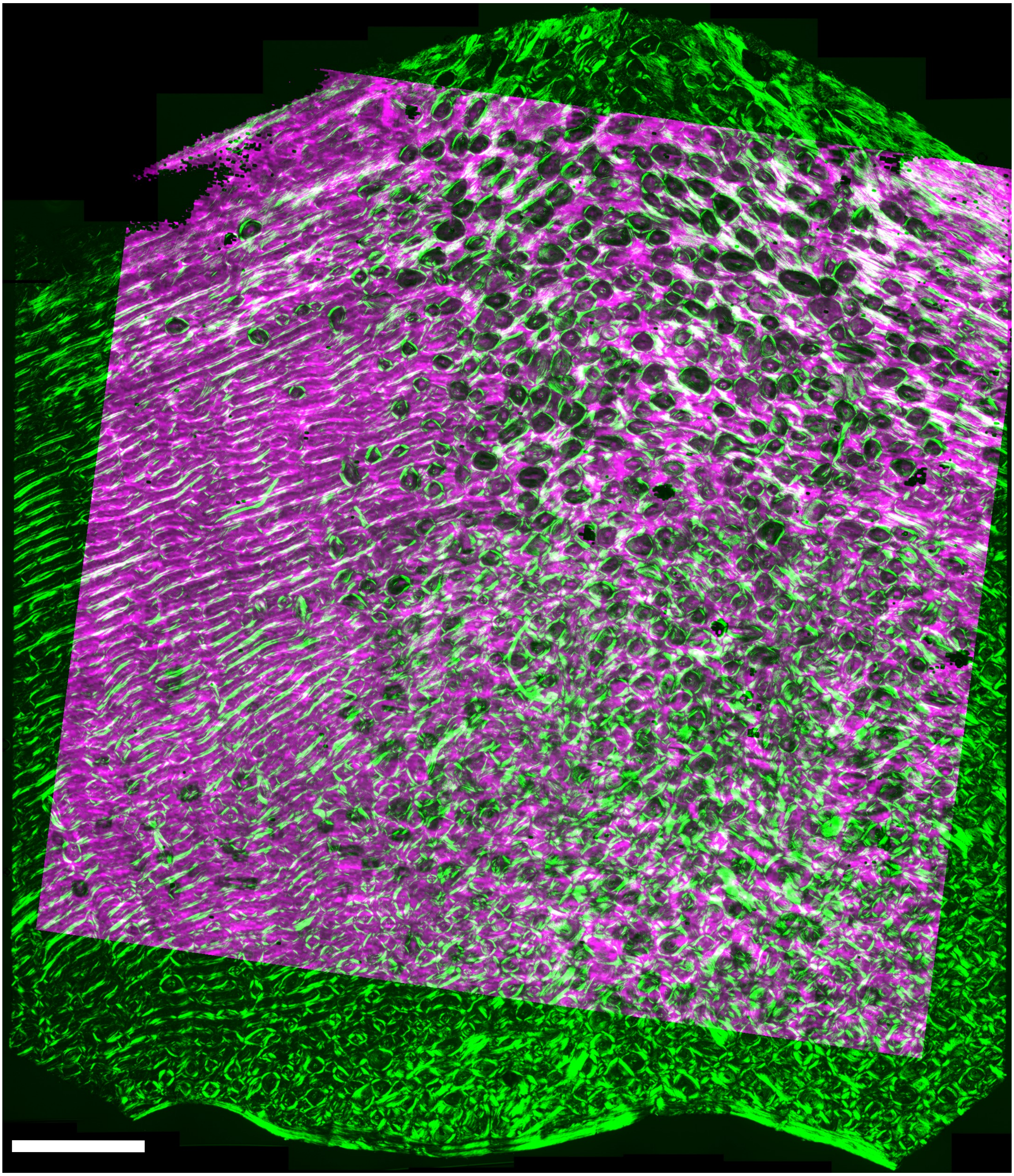

Supplement: S4 Fig — Overlay of the qsSAXSI image of 2π/α (pink) and the polarized light microscopy image (green) of the reference sample in the posterior region. Note that lower values of 2π/α, indicating a reduced spatial extent of the regularity in nanoparticle packing, can be observed in osteonal bone. Scale bars: 1,5 mm. (PDF) [file pone.0176179.s004.pdf]
