# Supplementary material for: Nanoscale modifications in the early heating stages of bone are heterogeneous at the microstructural scale
Source: PLoS One. 2017 Apr 19;12(4):e0176179. doi: 10.1371/journal.pone.0176179 (PMC5397064; doi:10.1371/journal.pone.0176179)

**S5 Fig. Site-matched correspondence between the tissue microstructure and tissue organization.** Overlay of the qsSAXSI image of $2\pi/\beta$ (blue) and the polarized light microscopy image of the reference sample in the posterior region. Note that the lower values of $2\pi/\beta$ indicating a closer nanoparticle packing correspond to the osteons and higher ones to interestitial tissue in the osteonal zone. Scale bars: 1,5 mm.

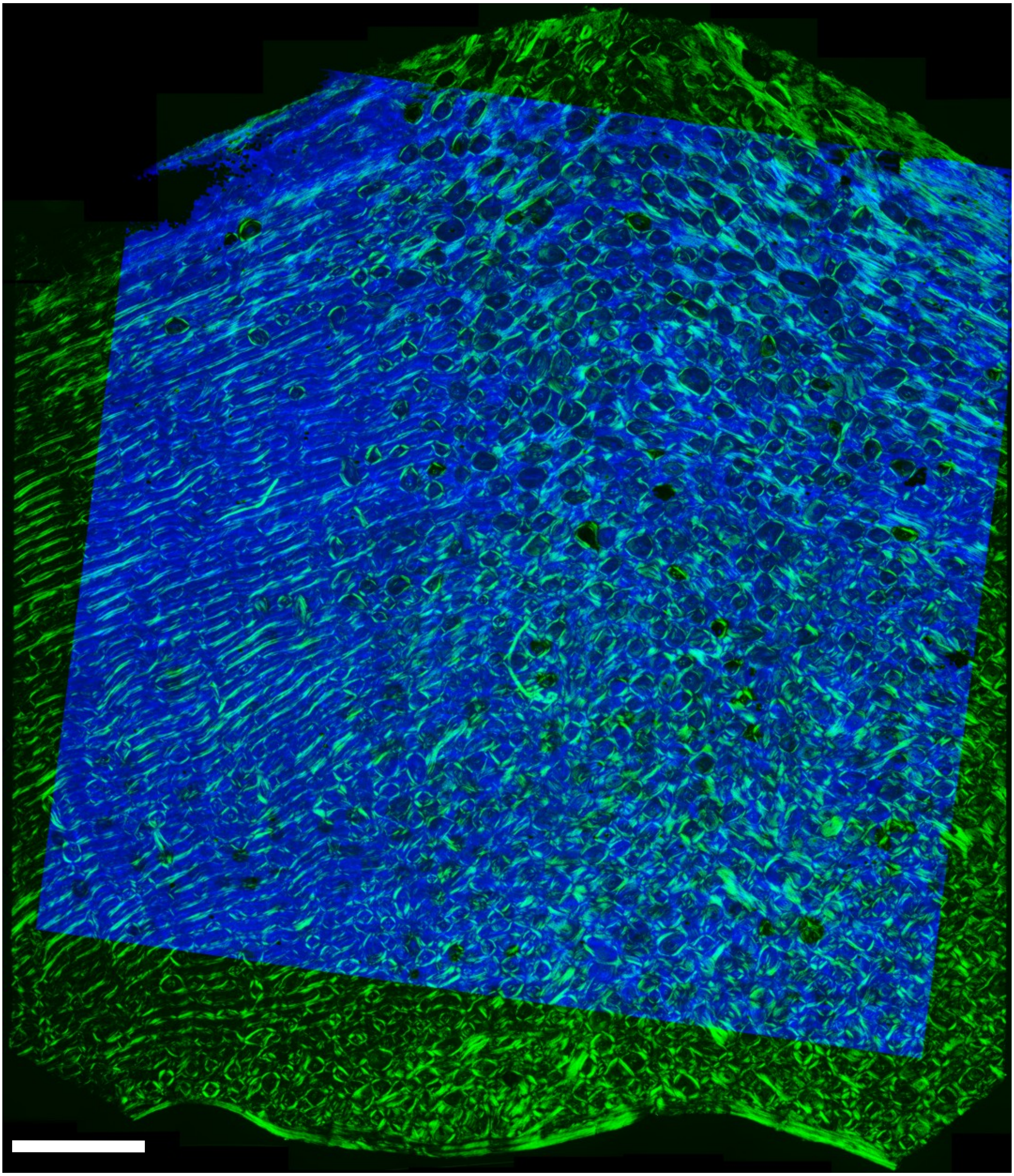

Supplement: S5 Fig — Overlay of the qsSAXSI image of 2π/β (blue) and the polarized light microscopy image of the reference sample in the posterior region. Note that the lower values of 2π/β indicating a closer nanoparticle packing correspond to the osteons and higher ones to interestitial tissue in the osteonal zone. Scale bars: 1,5 mm. (PDF) [file pone.0176179.s005.pdf]
