# Supplementary material for: Nanoscale modifications in the early heating stages of bone are heterogeneous at the microstructural scale
Source: PLoS One. 2017 Apr 19;12(4):e0176179. doi: 10.1371/journal.pone.0176179 (PMC5397064; doi:10.1371/journal.pone.0176179)
Supplement: S1 Table — (p-values and estimated 90% confidence interval for the difference between two populations). (PDF) [file pone.0176179.s006.pdf]

**S1 Table - $\nu_1 PO_4$**	*p-value*	*confidence interval*				
	150 ^{o}C		190 ^{o}C		210 ^{o}C	
Ref	0.143	**/**	**0.002**	**0.15 – 0.48**	**0.004**	**0.12 – 0.48**
150 ^{o}C			0.052	0.03 – 0.30	0.075	0.48 – 0.15
190 ^{o}C					0.970	**/**
