# Supplementary material for: Nanoscale modifications in the early heating stages of bone are heterogeneous at the microstructural scale
Source: PLoS One. 2017 Apr 19;12(4):e0176179. doi: 10.1371/journal.pone.0176179 (PMC5397064; doi:10.1371/journal.pone.0176179)
Supplement: S2 Table — (PDF) [file pone.0176179.s007.pdf]

S2 Table - ν_1CO_3 *p-value* *confidence interval*

	150 °C		190 °C		210 °C	
Ref	0.218	/	0.796	/	0.739	/
150 °C			0.190	/	0.123	/
190 °C					0.796	/
