# Supplementary material for: Nanoscale modifications in the early heating stages of bone are heterogeneous at the microstructural scale
Source: PLoS One. 2017 Apr 19;12(4):e0176179. doi: 10.1371/journal.pone.0176179 (PMC5397064; doi:10.1371/journal.pone.0176179)
Supplement: S3 Table — (PDF) [file pone.0176179.s008.pdf]

S3 Table - νAmide I *p-value* *confidence interval*

	150 °C		190 °C		210 °C	
Ref	0.105	**/**	0.190	**/**	**0.001**	**-3.22 – -1.11**
150 °C			0.739	**/**	**0.028**	**-2.33 – -0.46**
190 °C					**$<10^{-3}$**	**-2.11 – -0.89**
