# Supplementary material for: Nanoscale modifications in the early heating stages of bone are heterogeneous at the microstructural scale
Source: PLoS One. 2017 Apr 19;12(4):e0176179. doi: 10.1371/journal.pone.0176179 (PMC5397064; doi:10.1371/journal.pone.0176179)
Supplement: S4 Table — (PDF) [file pone.0176179.s009.pdf]

S4 Table - νCH *p-value* *confidence interval*

	150 °C		190 °C		210 °C	
Ref	0.063	**/**	0.218	**/**	0.143	**/**
150 °C			**0.001**	**-1.69 – -0.51**	**0.002**	**-2.36 – -0.75**
190 °C					0.579	**/**
