# Supplementary material for: Nanoscale modifications in the early heating stages of bone are heterogeneous at the microstructural scale
Source: PLoS One. 2017 Apr 19;12(4):e0176179. doi: 10.1371/journal.pone.0176179 (PMC5397064; doi:10.1371/journal.pone.0176179)
Supplement: S5 Table — (PDF) [file pone.0176179.s010.pdf]

S5 Table - ν_1CO_3 / ν_1PO_4 *p-value* *confidence interval*

	150 °C		190 °C		210 °C	
Ref	0.971	/	0.631	/	0.971	/
150 °C			0.684	/	0.971	/
190 °C					0.853	/
