# Supplementary material for: Nanoscale modifications in the early heating stages of bone are heterogeneous at the microstructural scale
Source: PLoS One. 2017 Apr 19;12(4):e0176179. doi: 10.1371/journal.pone.0176179 (PMC5397064; doi:10.1371/journal.pone.0176179)
Supplement: S6 Table — (PDF) [file pone.0176179.s011.pdf]

**S6 Table - ν_1PO_4 / νCH**	***p-value***	***confidence interval***				
	150 °C		190 °C		210 °C	
Ref	**<10^{-3}**	**2.90 – 6.64**	**<10^{-3}**	**3.87 – 6.32**	**<10^{-3}**	**3.92 – 6.91**
150 °C			0.529	**/**	0.353	**/**
190 °C					0.853	**/**
