# Supplementary material for: Nanoscale modifications in the early heating stages of bone are heterogeneous at the microstructural scale
Source: PLoS One. 2017 Apr 19;12(4):e0176179. doi: 10.1371/journal.pone.0176179 (PMC5397064; doi:10.1371/journal.pone.0176179)
Supplement: S7 Table — (PDF) [file pone.0176179.s012.pdf]

**S7 Table - TEM particle size**	*p-value*	*confidence interval*	*p-value*	*confidence interval*	*p-value*	*confidence interval*
	150 °C		170 °C		200 °C	
Ref	**<10^{-3}**	**2.22 – 4.98**	**<10^{-3}**	**2.12 – 4.56**	**0.017**	**0.49 – 1,45**
150 °C			0.959	**/**	**0.016**	**-3.54 – -0.88**
170 °C					**0.009**	**-3.28 – -0.97**
