# Supplementary material for: Nanoscale modifications in the early heating stages of bone are heterogeneous at the microstructural scale
Source: PLoS One. 2017 Apr 19;12(4):e0176179. doi: 10.1371/journal.pone.0176179 (PMC5397064; doi:10.1371/journal.pone.0176179)
Supplement: S8 Table — (PDF) [file pone.0176179.s013.pdf]

S8 Table - qsSAXSI particle size *p-value* *confidence interval*

	2		3		4		5		6	
1	**$<10^{-6}$**	**-0.04 – -0.03**	**$<10^{-6}$**	**-0.11 – -0.10**	**$<10^{-6}$**	**-0.18 – -0.17**	**$<10^{-6}$**	**-0.18 – -0.17**	**$<10^{-6}$**	**-0.18 – -0.17**
2			**$<10^{-6}$**	**-0.08 – -0.07**	**$<10^{-6}$**	**-0.14 – -0.13**	**$<10^{-6}$**	**-0.14 – -0.13**	**$<10^{-6}$**	**-0.14 – -0.13**
3					**$<10^{-6}$**	**-0.07 – -0.06**	**$<10^{-6}$**	**-0.07 – -0.06**	**$<10^{-6}$**	**-0.07 – -0.06**
4							0.747	**/**	0.634	**/**
5									0.846	**/**
